# The circadian clock of the bacterium *B. subtilis* evokes properties of complex, multicellular circadian systems

**DOI:** 10.1126/sciadv.adh1308

**Published:** 2023-08-04

**Authors:** Francesca Sartor, Xinming Xu, Tanja Popp, Antony N. Dodd, Ákos T. Kovács, Martha Merrow

**Affiliations:** ^1^ Institute of Medical Psychology, Medical Faculty, LMU Munich, Munich, Germany.; ^2^ Bacterial Interactions and Evolution Group, DTU Bioengineering, Technical University of Denmark, Kongens Lyngby, Denmark.; ^3^ Institute of Biology Leiden, Leiden University, Leiden, Netherlands.; ^4^ Department of Cell and Developmental Biology, John Innes Centre, Norwich Research Park, Norwich, UK.

## Abstract

Circadian clocks are pervasive throughout nature, yet only recently has this adaptive regulatory program been described in nonphotosynthetic bacteria. Here, we describe an inherent complexity in the *Bacillus subtilis* circadian clock. We find that *B. subtilis* entrains to blue and red light and that circadian entrainment is separable from masking through fluence titration and frequency demultiplication protocols. We identify circadian rhythmicity in constant light, consistent with the Aschoff’s rule, and entrainment aftereffects, both of which are properties described for eukaryotic circadian clocks. We report that circadian rhythms occur in wild isolates of this prokaryote, thus establishing them as a general property of this species, and that its circadian system responds to the environment in a complex fashion that is consistent with multicellular eukaryotic circadian systems.

## Teaser

Characteristics of complex circadian organization are exemplified in a non-photosynthetic prokaryote.

## INTRODUCTION

Circadian clocks are complex intracellular molecular networks that structure processes over the 24-hour day. They are commonly described as having self-sustained, temperature-compensated daily rhythms that entrain to 24-hour cycles of cycling environmental time cues (zeitgebers). These endogenous timing mechanisms are pervasive throughout nature; they operate from the level of cell to organism and from mammals, plants, and fungi to bacteria. Within the prokaryotic kingdom, which accounts for over 10% of life on Earth (*1*), a powerful clock model system in the cyanobacteria has been elaborated. Circadian clocks in nonphotosynthetic bacteria have only been described recently, and their characteristics are not well known (*2*–*5*). We and others recently showed evidence of the “trilogy” of circadian clock properties described above in *Bacillus subtilis* (*5*) and *Klebsiella aerogenes* (*4*).

However, there are many additional characteristics of circadian systems. In particular, probing the clock model organism with zeitgebers of different structures and strengths can reveal characteristic responses that are shared across a variety of model organisms (table S1). Imposing a zeitgeber cycle with a harmonic of 24 hours (so-called T cycles of, e.g., 12 or 8 hours) results in a frequency demultiplication, which manifests as 24-hour rhythms superimposed upon the shorter T cycle. This demonstrates the robustness of the circadian oscillation and the distinction from a driven response in the T cycle. The nature of a zeitgeber treatment itself can alter the free-running period (FRP) during subsequent free-running conditions, with these alterations called aftereffects. The Aschoff’s rule extends this principle: It describes the systematic lengthening or shortening of the FRP in constant light of increasing fluence rate (*6*). It would be naïve to assume that a prokaryotic circadian clock shares these properties with multicellular organisms. The characteristics that are shared and those that diverge relative to other clock systems would, presumably, hold insights into the mechanism and adaptive functions of the clock in nonphotosynthetic prokaryotes, compared with those in the eukaryotic and cyanobacterial clades.

In this study, we challenge the circadian clock of *B. subtilis* with respect to a catalog of chronobiology protocols. We use light as a zeitgeber to systematically probe the clock in this nonphotosynthetic bacterium. We found that this organism shares many circadian characteristics occurring in eukaryotic organisms, some of which have yet to be documented in established clock models in cyanobacteria or fungi.

## RESULTS

### Wild *B. subtilis* isolates exhibit circadian rhythms

Using the promoter region of the *ytvA* and *kinC* genes (encoding a blue light photoreceptor and a kinase, respectively) fused to the bacterial luciferase cassette, we identified circa 24-hour rhythms in bioluminescence in constant darkness that entrain to light or temperature cycles and are temperature compensated (*5*). In our previous experiments, we used a laboratory strain of *B. subtilis* (strain 168) and rhythms were observed only in cultures that formed biofilm. Domesticated strains are generally distinct from true wild types and they can lack certain regulatory pathways present in natural accessions (*7*). In the case of strain 168, biofilm development is altered (*8*) and it harbors a mutation that prevents swarming behavior (*9*).

**Fig. 1.**
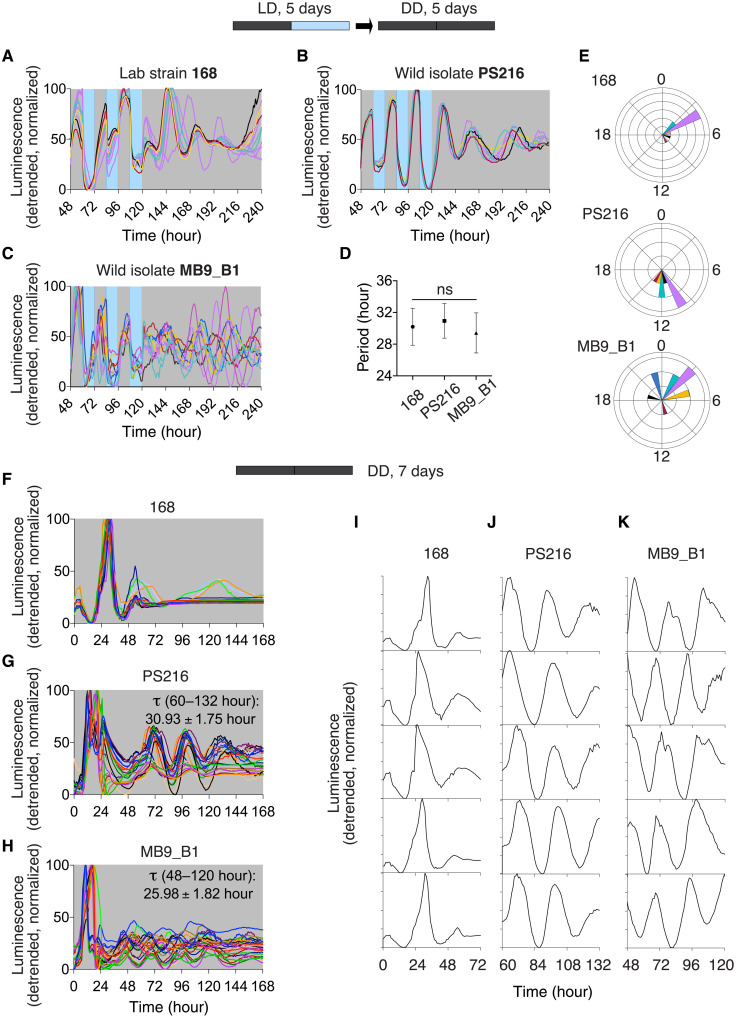
Circadian rhythms occur in *B. subtilis* laboratory and wild-isolated strains. (**A** to **E**) Circadian rhythm in *ytvA* promoter expression in DD following entrainment with bLD cycles. The strains P*_ytvA_-lux* 168 (A) (*n* = 10), P*_ytvA_-lux* PS216 (B) (*n* = 8), and P*_ytvA_-lux* MB9_B1 (C) (*n* = 11) were incubated in bLD cycles for 5 days and then released to DD. Gray and blue backgrounds correspond to dark and light incubations, respectively. Baseline-detrended, normalized luminescence is shown. (D) Mean FRP was determined for hours 123 to 195 (ns, *P* > 0.05 for all strains; one-way ANOVA followed by Tukey’s multiple comparison test). Error bars represent SD. (E) Polar plots showing the phase of the first bioluminescence peak on the first day of release to DD. The color of the phases in the polar plot matches the corresponding bioluminescence traces in (A) to (C). (**F** to **K**) Circadian rhythm in *ytvA* promoter expression in cultures maintained in DD from their inoculation. The strains P*_ytvA_-lux* 168 (F) (*n* = 17), P*_ytvA_-lux* PS216 (G) (*n* = 24), and P*_ytvA_-lux* MB9_B1 (H) (*n* = 26) were incubated in DD for 7 days. Baseline-detrended, normalized luminescence is shown. FRP and the time window for period calculation is reported on the graphs. Representative single traces are shown in (I) to (K). Bacteria were grown at a constant temperature of 27°C.

We wished to determine whether circadian behavior in *B. subtilis* is a general phenomenon, or a peculiarity of strain 168. We transformed the luciferase construct for monitoring *ytvA* promoter activity (P*_ytvA_-lux*) into two wild isolates: PS216 (isolated from sandy soil in Slovenia) (*10*) and MB9_B1 (isolated from the hyphosphere of an *Agaricus* sp. fungus from Denmark) (*11*). Both of these strains are capable of forming robust biofilms, and they have intact swarming behavior. We incubated the lab strain (168, P*_ytvA_-lux*) and the wild isolates (PS216, P*_ytvA_-lux*; MB9_B1, P*_ytvA_-lux*) for 5 days in cycles of 12-hour blue light and 12-hour darkness (24-hour bLD), after which they were released into conditions of constant darkness and temperature (DD; Fig. 1, A to C). Rhythmic promoter activity occurred in all strains, suggesting that circadian rhythms and their ability to entrain using light cycles are a general property in *B. subtilis*, rather than being restricted to a single laboratory derivate.

**Table 1.** Strains and plasmids used in this study.

Strain (short name)	Genotype	Source
168	*sacA*::P*_ytvA_*-*luxABCDE* (Cm^R^)	(*5*)
MB9_B1	*sacA*::P*_ytvA_*-*luxABCDE* (Cm^R^)	This study
PS216	*sacA*::P*_ytvA_*-*luxABCDE* (Cm^R^)	This study
BKK30340	∆*ytvA*::Km^R^	(*66*)
BKK34110	*∆rsbP*::Km^R^	(*66*)
BKK34100	*∆rsbQ*::Km^R^	(*66*)
BKK04730	*∆sigB*::Km^R^	(*66*)
PS216	*∆ytvA*::Km^R^*, sacA*::P*_ytvA_*-*luxABCDE* (Cm^R^)	This study
PS216	*∆rsbP*::Km^R^*, sacA*::P*_ytvA_*-*luxABCDE* (Cm^R^)	This study
PS216	*∆rsbQ*::Km^R^*, sacA*::P*_ytvA_*-*luxABCDE* (Cm^R^)	This study
PS216	*∆sigB*::Km^R^*, sacA*::P*_ytvA_*-*luxABCDE* (Cm^R^)	This study
PS216	*∆ytvA*::Km^R^*, ∆rsbP*::Km^R^*, sacA*::P*_ytvA_*-*luxABCDE* (Cm^R^)	This study
PS216	*∆ytvA*::Km^R^*, ∆rsbQ*::Km^R^*, sacA*::P*_ytvA_*-*luxABCDE* (Cm^R^)	This study
PS216	*∆ytvA*::Km^R^*, ∆sigB*::Km^R^*, sacA*::P*_ytvA_*-*luxABCDE* (Cm^R^)	This study
PS216 (TB269)	*amyE:P_hy_-gfp*	(*67*)
Plasmid pMH66	pNZ124-based Cre-encoding plasmid, Tet^R^ Ts	(*68*)

The FRP was not significantly different between the strains tested [168, 30.20 ± 2.33 hours; PS216, 30.94 ± 2.19 hours; and MB9_B1, 29.42 ± 2.52 hours; not significant, *P* > 0.05, one-way analysis of variance (ANOVA) followed by Tukey’s multiple comparison test; Fig. 1D]. Individual MB9_B1 cultures were less synchronized following this entrainment protocol relative to the other two strains, as indicated by a higher SD of the mean of the first peak after release into DD (MB9_B1, 9.18 ± 8.90 hours; 168, 4.80 ± 2.15 hours; and PS216, 11.50 ± 1.51 hours). The polar diagrams in Fig. 1E depict the broader distribution in phases upon release to DD in the MB9_B1 cultures than in the 168 and PS216 cultures.

**Table 2.** Primers used in this study.

Primer	Sequence (5′ → 3′)	Target locus
oXM1	CTTCATCATCACCTTCCT	*ytvA*
oXM2	GGGCTCTTGTTTTATCTCT	*ytvA*
oXM3	ACACTCTTCAAACCATCC	*rsbQ*
oXM4	GGTCACCTCTATCCCTTT	*rsbQ*
oXM5	GTCTGAAACAAATGGAGG	*rsbP*
oXM6	CAGGTGTTTCAATATGCG	*rsbP*
oXM7	TTAATGGAAACGCTCATGG	*sigB*
oXM8	TGCTTTCTACGTCTTCAC	*sigB*

All rhythmic samples of all strains formed a floating biofilm at the liquid-air interface of the cultures, consistent with our previous observations (*5*). Given the association of rhythmicity and biofilm formation, and given that biofilm forms in these cultures in the absence of zeitgeber cycles, the emergence of circadian rhythms was examined in cultures that developed in constant darkness and temperature, where no apparent zeitgebers were present during the initial biofilm development. Unexpectedly, all strains exhibited emergent circadian rhythms (Fig. 1, F to H; representative, single cultures are shown in Fig. 1, I to K; all traces of rhythmic samples are shown in fig. S1). In 168, rhythms in DD-grown cultures damped rapidly within the first 3 days, precluding a rigorous analysis of the FRP, whereas PS216 and MB9_B1 cultures had sustained circadian rhythms that persisted for about 5 days. For the 168 strain, 53.13% of the samples were rudimentarily rhythmic in the first 3 days, whereas 75% of the PS216 cultures were rhythmic with a period of 30.93 ± 1.75 hours and 83.87% of the MB9_B1 cultures showed rhythms with a period of 25.98 ± 1.82 hours.

The local environment in the 96-well plate cultures will constantly change over the course of the experiment. This includes nutrient use and metabolite secretion, as will occur also for circadian studies in other systems such as tissue culture, plants and cyanobacteria. Given that the waveform can change over the course of an experiment [as is common in many other circadian systems (*3*, *12*–*14*)], we wished to verify that the cultures remained viable throughout the experiment. We examined total cell number of PS216 cultures by counting CFUs from sonicated biofilms (fig. S2A) and by using a constitutive green fluorescent protein (GFP) fluorescence promoter reporter (fig. S2B). During the experiment, total cell number steadily increased and cultures remained viable in constant conditions.

### Systematic assessment of circadian entrainment to light as a zeitgeber in *B. subtilis*

**Fig. 2.**
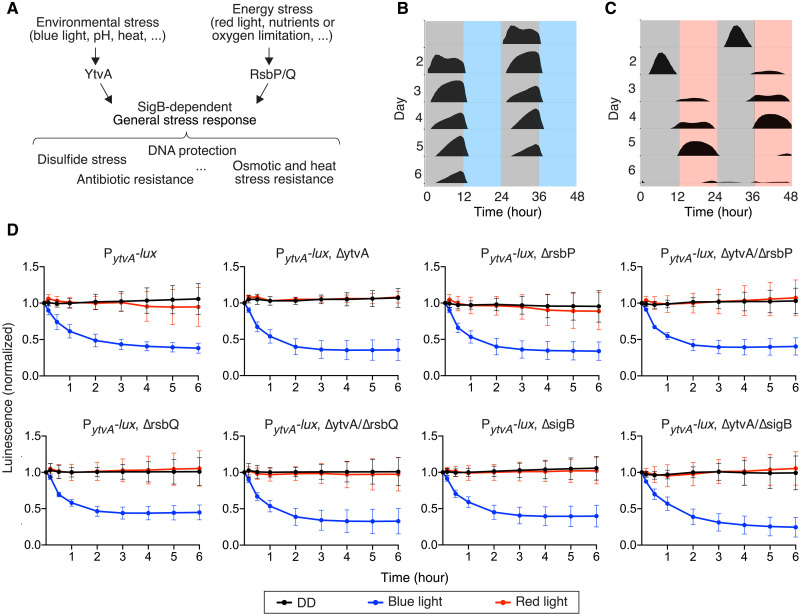
Entrainment and masking to spectrally defined light. (**A**) The pathways leading to activation of the σ^B^-dependent general stress response upon exposure to stress stimuli, including blue and red light. (**B** and **C**) Double plots show reporter gene expression under entrainment to bLD (B) (*n* = 18) or rLD (C) (*n* = 27) cycles starting from the second day of incubation. Fluence rates were 15 μE m^−2^ s^−1^. Red, blue, and gray areas indicate the red light, blue light, and dark phases, respectively, of the zeitgeber cycles. (**D**) A masking assay shows the acute response to spectrally defined illumination. Bioluminescence traces of P*_ytvA_-lux* PS216 are shown, normalized to the *t* = 0 time point (48 hours of growth). Host strains are wild-type, knockouts of *ytvA*, *rsbP*, *rsbQ*, *sigB*, and double knockouts *ytvA/rsbP*, *ytvA/rsbQ*, and *ytvA/sigB*. The mean signal from 12 wells ±SD is shown. Red and blue light fluence rates were 30 μE m^−2^ s^−1^. When comparing blue light versus DD, the signal was significantly different from 30 min to 6 hours (*P* < 0.0001) for all genotypes. For the genotype ∆*ytvA/*∆*rsbQ*, the signal was significantly different after 10 min (*P* < 0.05) (two-way ANOVA followed by Dunnett’s multiple comparison test).

Entrainment to zeitgeber cycles is what determines the circadian phase (*15*), which in turn is adaptive because it defines the temporal positioning of biological processes. We used light as a zeitgeber to systematically explore entrainment properties in *B. subtilis*. We selected the strain PS216 for all further experiments here because of the combination of self-sustained rhythms and tighter phase control through entrainment in 24-hour bLD cycles (Fig. 1E and as discussed above). *B. subtilis* is reported to be photoreceptive to both blue and red light (*16*) with both wavelengths activating the general stress response pathway, albeit via alternative routes (Fig. 2A). Entrainment of *B. subtilis* using 24-hour blue- or red-light cycles was compared. Under bLD cycles, the P*_ytvA_-lux* bioluminescence signal peaks in the middle of the dark phase (Fig. 2B), whereas under red LD (rLD) cycles, it shifts to the light phase (Fig. 2C). The entrained phase of the luminescence signal (center of gravity; fig. S3), calculated between days 3 and 5 in reference to midnight, was 2.97 ± 0.90 hours for samples in bLD cycles and 9.99 ± 1.20 hours for samples in rLD cycles.

The bioluminescence traces of cultures in 24-hour bLD cycles (Figs. 1, A to C, and 2B) were consistent between all strains, in that *ytvA* promoter activity increased during the dark phase and decreased abruptly during the light phase. Blue light appears to acutely suppress *ytvA* promoter activity in a process that resembles masking, a noncircadian response of the organism to a zeitgeber of the circadian clock. Masking is not incompatible with the presence of a functional clock and can also be modulated by the clock (*17*–*20*). We developed an assay based on *ytvA* promoter activity to quantify masking by red or blue light (Fig. 2D). PS216 cultures expressing the P*_ytvA_-lux* reporter were grown in 96-well plates in darkness for 48 hours, at which time they were exposed to illumination with either blue or red light. Control plates were kept in darkness. Bioluminescence was measured at 10 and 30 min, 1 hour, 2 hours, 4 hours, and 6 hours into the light exposure. Within 30 min of illumination with blue light, P*_ytvA_-lux* bioluminescence was down-regulated significantly (*P* < 0.0001; two-way ANOVA followed by Tukey’s multiple comparison test; Fig. 2D). This down-regulation was confirmed at the level of *ytvA* transcript abundance (fig. S4). In contrast, red light did not significantly modify reporter activity relative to the control samples held in darkness (*P* > 0.05; two-way ANOVA followed by Tukey’s multiple comparison test; Fig. 2D). Therefore, the calculated phases for entrainment using blue but not red light are modified by masking. These results indicate that *B. subtilis* responds to blue and red light in a very distinct way.

Informed by the regulatory architecture of light signaling in *B. subtilis* (Fig. 2A), we assayed knockout mutants of the blue light photoreceptor YtvA, the putative blue- and red-light-responsive proteins RsbP/Q, and the stress-responsive σ factor SigB for the masking of P*_ytvA_-lux* in response to blue light. We hypothesized that these proteins would be involved in phototransduction and masking shown by the *ytvA* promoter expression. Unexpectedly, as in the wild-type background, P*_ytvA_-lux* expression was down-regulated in response to illumination with blue light in all of these mutants (Fig. 2D). Double mutants for *ytvA*/*rsbP*, *ytvA*/*rsbQ*, and *ytvA*/*sigB* also have responses to blue light that are comparable to the parental strain (Fig. 2D). This suggests that *B. subtilis* has additional, unidentified blue-light-photosensitive molecules.

**Fig. 3.**
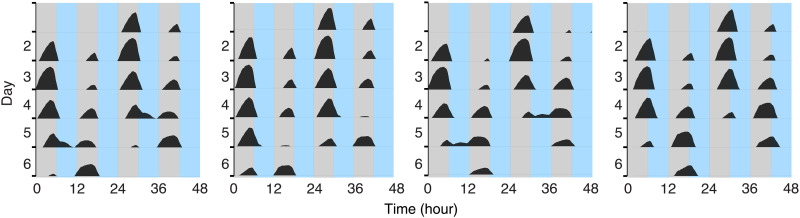
Frequency demultiplication occurs in *B. subtilis* under 12-hour zeitgeber cycles. PS216 cultures expressing the P*_ytvA_-lux* reporter were incubated for 6 days in 12-hour bLD cycles (6-hour darkness/6-hour light at 30 μE m^−2^ s^−1^; *n* = 22). Four representative samples are shown here. All samples are shown in fig. S5. Profiles correspond to smoothed, detrended, bioluminescence traces of the P*_ytvA_-lux.* Data are double plotted; only positive values are shown.

With such a robust masking response, how can we dissect whether the clock of *B. subtilis* entrains in bLD cycles or, alternatively, whether the rhythmic reporter gene expression is a “driven” response to the environmental stimuli? To distinguish between masking and entraining components in response to blue light, we applied T cycles with a duration that is a divisor of the typical (24 hours) zeitgeber cycle (*21*–*23*). Such cycles lead to a frequency demultiplication of the circadian system (*21*), identified by the appearance of a major, circadian component once per 24 hours (i.e., in alternate cycles) compared to a minor, masking component in the other cycles. A purely driven response would show equivalent signals each 12-hour zeitgeber cycle. The P*_ytvA_-lux* PS216 strain was held in 12-hour bLD cycles (6/6 hours, 30 μE m^−2^ s^−1^) yielding a nonsymmetrical pattern of promoter activity. There was a dominant peak in alternate cycles (i.e., once per 24 hours; Fig. 3 and fig. S5). The dominant peak moved to the opposite cycle after 4 to 5 days in most samples (fig. S5). This is consistent with the entrainment properties “range of entrainment” and “relative coordination” (*21*): the bacterial clock, which has a FRP longer than 24 hours, briefly entrains before eventually breaking free and jumping to the next 12-hour cycle. These patterns are consistent with the coexistence of a circadian clock and a masking component occurring additively as represented in the larger peaks, and only a masking component in the minor ones. Therefore, *B. subtilis* integrates circadian and noncircadian responses to light, and these two processes can be distinguished. This is similar to the dynamics of eukaryotic circadian systems (*22*).

**Fig. 4.**
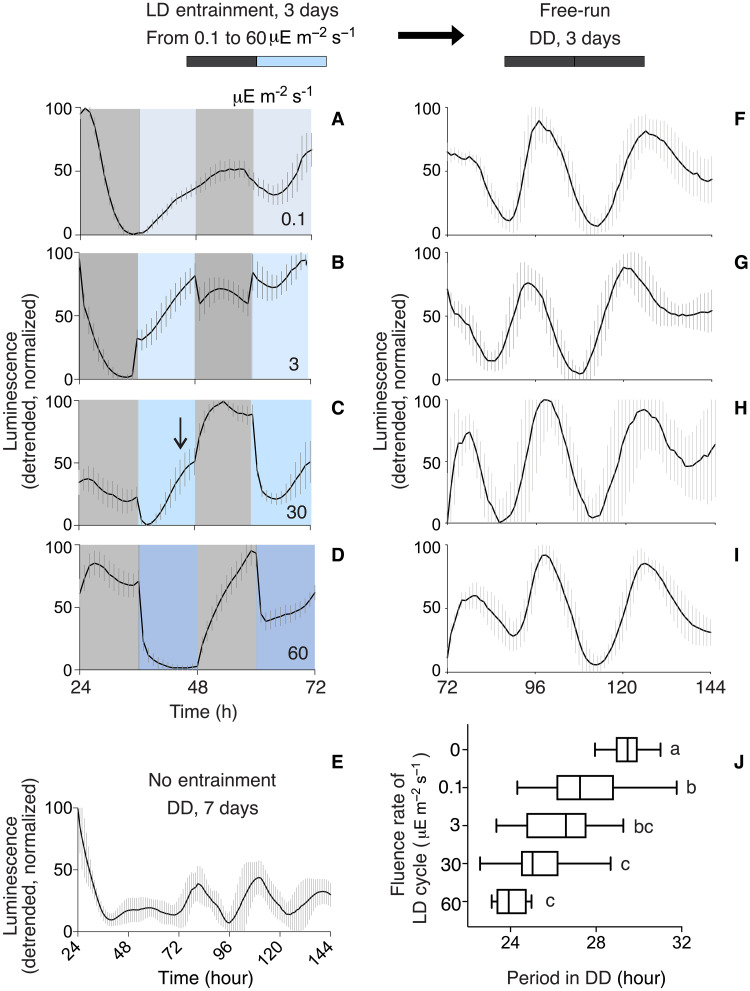
Titration of blue light in an entrainment protocol shows aftereffects and loss of masking. (**A** to **D**) Phase of entrainment changes and masking decreases with zeitgeber strength. The strain P*_ytvA_-lux* PS216 was incubated in bLD cycles of different fluence rates for 3 days (12-hour dark/12-hour light). P*_ytvA_-lux* reporter expression is shown from 24 to 72 hours of incubation. Light intensities shown are 0.1 to 60 μE m^−2^ s^−1^. Blue and gray areas indicate the blue light and the dark phases, respectively. Number of samples included in (A) and (F), 30; in (B) and (G), 11; in (C) and (H), 17; and in (D) and (I), 14. Reporter activity was further recorded in cultures which never experienced zeitgeber cycles (**E**) (*n* = 18). (**F** to **J**) FRP changes according to the zeitgeber strength of the previous entraining bLD cycles. After 3 days in 12/12-hour bLD conditions, reporter gene activity was recorded for 3 days in DD [(F) to (I)]. (A) to (I) The averaged, linear-detrended, normalized bioluminescence traces with SD are shown. Temperature was kept constant at 27°C. (J) The FRP of the rhythmic samples was calculated. Data distribution is shown using box blots with Tukey whiskers. Letters denote significantly different FRPs between conditions (see also table S2).

In many organisms including humans, entrained phase changes with zeitgeber strength (*15*). We wished to determine whether this is also the case in nonphotosynthetic bacteria. P*_ytvA_-lux* PS216 was incubated in 12:12-hour bLD cycles with fluence rates ranging from 0.1 to 60 μE m^−2^ s^−1^. At light levels lower than 60 μE m^−2^ s^−1^, masking dissipated (Fig. 4, A to C, compared to Fig. 4D). The phase of bioluminescence expression occurs earlier (advances) in 30 μE m^−2^ s^−1^ light levels, as indicated by the appearance of a shoulder of gene expression in blue light (Fig. 4C, see arrow). At 3 μE m^−2^ s^−1^, the reporter construct transitioned from suppression to induction in light. The promoter expression is modestly repressed by the onset of darkness at this fluence rate. At 0.1 μE m^−2^ s^−1^, no masking occurred, yet the *B. subtilis* clock appears to be synchronizing to this extremely low light zeitgeber cycle based on a comparison with the bioluminescent traces of DD cultures (fig. S6). We also note fewer rhythmic samples when cultured entirely in DD compared to those from bLD cycles of all fluence rates (% of rhythmic samples in DD: 47.36%; and in bLD cycles of 0.1, 3, 30, 60 μE m^−2^ s^−1^: 96.77, 100, 94.44, and 100%). Therefore, 24-h light cycles support the development of a competent circadian system over a broad range of light conditions and entrainment patterns.

Aftereffects can occur in circadian systems, whereby the FRP varies systematically according to previous zeitgeber treatments (*24*, *25*). We calculated the FRP in DD, following entrainment in LD cycles of different fluence rates. Cultures entraining to higher fluence rates had a shorter FRP (closer to that of the entraining cycle) than those entraining in a lower amplitude zeitgeber cycle (Fig. 4, F to I and J ; FRP after DD and LD cycles of 0, 0.1, 3, 30, 60 μE m^−2^ s^−1^: 29.60 ± 1.32 hours, 27.45 ± 1.89 hours, 26.35 ± 1.82 hours, 25.36 ± 1.65 hours, and 24.07 ± 0.68 hours). The DD condition was significantly different from all other light conditions, as was 0.1 μE m^−2^ s^−1^ when compared with 30 and 60 μE m^−2^ s^−1^ (Fig. 4J and table S2). The systematic change in the FRP based on the fluence rate (hence, amplitude) used in the entraining cycle is consistent with the phenomenon of aftereffects of entrainment, indicating that the circadian system of *B. subtilis* stores information concerning previous environmental conditions.

**Fig. 5.**
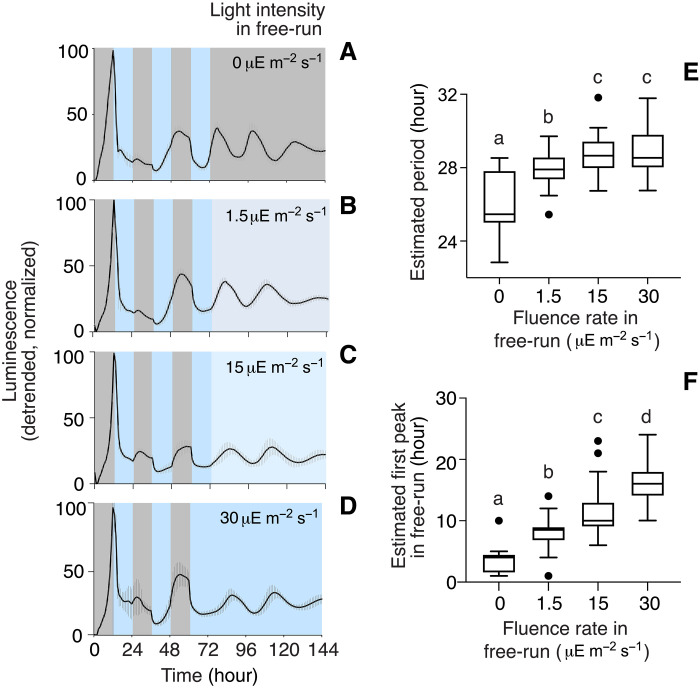
*B. subtilis* is rhythmic in constant light; period and phase are regulated by fluence rate. (**A** to **D**) Bioluminescence traces in entrainment and constant conditions of different fluence rates. P*_ytvA_-lux* PS216 was incubated in bLD cycles for 3 days (12-hour dark/12-hour light; 30 μE m^−2^ s^−1^) and then released to DD (A) or LL [(B) to (D); fluence rates shown on graphs] for 72 hours. Gray and blue backgrounds correspond to dark and light incubations, respectively. The averaged, linear-detrended, normalized bioluminescence traces with SD are shown. Temperature was kept at 27°C. (**E**) FRP lengthens with increasing fluence rate. The FRP during the first 3 days of LL was calculated. Data distribution is shown using box blots with Tukey whiskers. (**F**) Phase delay upon release in constant conditions with increasing fluence rate. Timing of the first peak following release in constant darkness or light was calculated. (E) and (F) Analysis included two biological replicates for each light condition. Number of samples included: DD, 25; 1.5 μE m^−2^ s^−1^, 30; 15 μE m^−2^ s^−1^, 59; and 30 μE m^−2^ s^−1^, 44. One representative replicate is shown in (A) to (D). (E) and (F) Different letters indicate significant differences between pairwise comparisons (see also table S2 and data file S1).

Another systematic response of circadian clocks is the Aschoff’s rule (*6*). As light levels increase, the FRP in constant light (LL) generally either increases (nocturnal organisms) or decreases (diurnal organisms; exceptions have been noted). Furthermore, it is not uncommon for rhythms to be suppressed in constant light. We determined the FRP of *B. subtilis* P*_ytvA_-lux* PS216 in DD or in LL at several different fluence rates following 3 days of entrainment in a 24-hour bLD cycle (30 μE m^−2^ s^−1^). For each fluence rate (0, 1.5, 15 and 30 μE m^−2^ s^−1^), two datasets from two independent experiments were combined within the analysis using a linear mixed modeling approach (data file S1) (*26*). *B. subtilis* was rhythmic in both DD and in all LL conditions tested (Fig. 5, A to D). The linear mixed model revealed a significant lengthening of the estimated period for cultures in LL in comparison to DD (Fig. 5E, table S2, and data file S1). In addition, the first peak of reporter gene activity in constant conditions was delayed significantly when cultures were released to increasing fluence rates of LL (Fig. 5F, table S2, and data file S1), consistent with the idea that the phase is related to the period.

## DISCUSSION

Evidence for circadian clocks in nonphotosynthetic prokaryotes has been limited, despite overwhelming evidence that circadian clock mechanisms are pervasive across nature. Building on the identification of circadian clocks in two species of nonphotosynthetic bacteria [*K. aerogenes* (*3*, *4*) and *B. subtilis* (*5*)], here, we investigated *B. subtilis* for circadian clock properties typically observed in complex organisms. The list of properties and principles established here includes the extension of circadian rhythms to wild-type isolates of *B. subtilis*, the emergence of circadian rhythms in constant conditions (DD) without previous zeitgeber cycles, differential circadian responses to blue and red light, and dissection of entrainment and masking properties to the blue light as zeitgeber. This last point includes frequency demultiplication in 12-hour bLD cycles, aftereffects, and changes in FRP based on fluence rate in constant conditions (Aschoff’s rule). Here, we will attempt to put these into context, especially with respect to other well-characterized circadian systems.

Entrainment leads to the establishment of a stable phase relationship between the external (environmental) and the internal (circadian) time. Circadian systems use zeitgebers for entrainment, leading to a set of remarkable phenomena. We were surprised to observe that a prokaryote challenged with chronobiological protocols exhibits a variety of highly complex entrainment properties. For instance, aftereffects describe changes in the FRP following specific zeitgeber treatments. Commonly used protocols revealing such changes include T cycles (entrainment cycles of different lengths) or treatments with various zeitgeber structures. The presence of aftereffects (see table S1) suggests that information regarding zeitgeber exposure is stored, much like a memory. In the case of *B. subtilis*, the zeitgeber treatment is present as the circadian system forms, from inoculation to biofilm formation. Aftereffects have also been reported in rodents that were exposed to exotic zeitgeber conditions throughout development (*27*). Discovering mechanisms by which this memory of entrainment conditions during development of a circadian system occurs, in diverse systems, will inform on convergent and divergent evolutionary processes.

The entrainment by the *B. subtilis* clock to different phases in blue light, red light, and temperature might seem discordant with the role of entrainment in establishing a particular phase relationship between the circadian clock and the day/night cycle. However, this phenomenon is not unique to *B. subtilis*. A notable example occurs for the circadian clock in *Lingulodinium polyedra* (previously called *Gonyaulax polyedra*) (*28*). Blue and red lights yield distinct phase response curves, indicating distinct entrained phases to each of these spectrally defined light sources. The light quality also determined the internal phase relationships of the flash and glow rhythms of *L. polyedra*, which are controlled by distinct circadian oscillators (*28*). The two oscillators regulating the flashing and the aggregation rhythms can be induced to run free with different FRPs by incubating in constant red light as opposed to white light, where they remain synchronized (*29*). Plants also use blue and red light photoreceptors for entrainment. Blue and red light sources can cause different entrained phases, depending on the phase of the light stimulus (*30*, *31*). Together with our findings, these reports open a number of testable hypotheses. First, blue and red light could entrain via their distinct energy profiles, producing different zeitgeber strengths. Second, blue and red light could entrain to distinct phases because they enter the system through distinct molecular substrates (see discussion below on photoreceptors). This will become clearer for *B. subtilis* once these pathways are defined. Third, in the case of *B. subtilis*, these entrainment characteristics might relate to its ecology. The spectral composition of light changes over the course of the day, as a function of weather conditions (*32*) and due to the local biotic and abiotic environment. For a soil bacterium such as *B. subtilis*, there might be a requirement to entrain differently to light levels and spectra that penetrate soils to different depths. There is evidence from *Arabidopsis* that light can be piped from the aerial parts of the plants to the roots (*33*, *34*); thus, *B. subtilis* associated with a plant microbiome would encounter additional possibilities for subterranean entrainment to light.

As with other organisms, the responsiveness of the entrained phase of *B. subtilis* to different spectral qualities of light suggests the functional relevance of the clock in *B. subtilis*. This further suggests an adaptive function, because the phase relationship between the clock and the environment confers benefits in other, phylogenetically distant organisms (e.g., cyanobacteria, plants, and animals) (*35*–*37*). Given the ecology of complex microbiomes, this responsive phasing may facilitate coordination of varied and local circadian programs within a microbiome (*38*), according to the abiotic and biotic environment. Our observations also underscore that a combination of zeitgebers is used by *B. subtilis*, which is analogous to the situation for fungal, mammalian, and plant cells. The task of the circadian clock is to “read” the local environment and, for many systems, this means harvesting not just one but many cues. We suggest that by using both blue and red light and temperature as zeitgebers, *B. subtilis* can fine-tune clock-regulated processes to a greater range of situations.

Although we had previously established that *B. subtilis* entrains in light-dark cycles (*5*), the light-sensing mechanisms used by *B. subtilis* for the purpose of entrainment remain unknown. In stationary phase cells, YtvA is activated by blue light and acts as positive regulator of the SigB-dependent general stress response pathway (*39*–*42*). When we tested strains carrying knockouts of the *ytvA* gene, cultures remained able to perceive blue light, as evidenced by an intact masking response (Fig. 2E). Additional blue light–sensing proteins in *B. subtilis* include the protein serine phosphatase RsbP together with the α/β-hydrolase RsbQ. RsbP/Q are primarily responsive to energy stresses and red light, and it has been shown that their activation can also induce σ^B^ activity upon blue light illumination, through a stress pathway independent of YtvA (*16*). However, we observed that reporter gene expression was down-regulated upon blue light illumination in the *rsbP*, *rsbQ*, and *sigB* single mutants and all double mutants combined with the *ytvA* knockout allele that were tested. Therefore, these data suggest that *B. subtilis* may have additional and unidentified phototransduction mechanisms. This could include participation of redox- or indirect metabolically mediated mechanisms. This is especially interesting given that circadian redox cycles have been reported in a variety of other phylogenetically distant organisms (*43*). For example, bacteria can sense light through nondedicated photoreceptor molecules. Fe/S clusters can absorb in the ultraviolet/visible part of the spectrum (*44*–*47*) and have been implicated in circadian clock input pathway of cyanobacteria (*48*). Light can also entrain the cyanobacterial circadian clock via metabolism, by toggling the adenosine 5′-triphosphate/adenosine 5′-diphosphate (ATP/ADP) ratio (*49*). Furthermore, *B. subtilis* contains riboflavins (vitamin B2) and heme (*47*, *50*), which are light sensitive. Future studies are required to investigate which light-sensing molecules are involved in entraining the *B. subtilis* circadian system, which might also provide insights into the ecological and adaptive relevance of circadian programs in *B. subtilis*.

Our study has a number of important implications. First, circadian clock properties are broadly found in *B. subtilis*, not simply in a laboratory strain. The requirement for biofilm formation for detection of rhythmicity remains consistent across strains. As biofilm is a highly organized structure encompassing differentiated cells, *B. subtilis* may serve as a model for the important but understudied concept of circadian organization. Circadian organization refers to a circadian system that is composed of rhythmic subunits, which, as a whole, must have mechanisms for orderly and choreographed synchronization. In plants and animals, we know that each cell has a circadian clock. Through cell-, organ-, and organism-specific coupling mechanisms, a nonchaotic synchrony is achieved (*51*–*54*).The emergence of synchronized oscillations that occurs in *B. subtilis* cultures in the absence of regular, external cues (Fig. 1, F to K) indicates that intrinsic processes of cell-cell communication within *B. subtilis* biofilms occur that are permissive and sufficient for sustained, rhythmic processes. We report period lengths that vary systematically but widely depending on the zeitgeber history, stressing the conditional nature of the FRP of *B. subtilis*. This is also true for other circadian systems (*55*, *56*) and we suggest that it reflects, on one hand, circadian organization (within the biofilm) and, on the other hand, the adaptive nature of entrained phase. This is important in the context of FRP, because this contributes to the entrained phase. Therefore, understanding circadian organization is critical because its disruption, for instance with shift work or jet lag, leads to loss of synchrony and decreased fitness or ill health (*57*, *58*). At present, we have only limited tools to study this problem of circadian organization in complex organisms. Our work emphasizes the intricacy of the connections between the zeitgeber integration system and the functioning of the entire circadian system (*59*, *60*). With a better understanding of how the inputs and the oscillator are interdependent, this knowledge can be applied to questions such as those concerning the importance of light entrainment to the consequences of chronopharmacology (*61*).

In conclusion, we find it remarkable that a relatively simple prokaryote, which lacks the obvious hierarchy of organization of multicellular organisms, evokes properties of complex circadian systems. This indicates that *B. subtilis* represents a powerful model system for the study of circadian clocks, given the scope of formal properties therein that it displays. It also tells us something about common elements of all circadian systems.

## MATERIALS AND METHODS

### Strains and strain construction

The strains used in this study are reported in Table 1. The sequences of the primers used in this study are provided in Table 2. The P*_ytvA_-lux* strains contain the promoter region of *ytvA* cloned in front of the promotor-less *luxABCDE* operon as generated in Eelderink-Chen *et al*. (*5*). All *B. subtilis* strains were constructed by natural competence transformation using genomic or plasmid DNA. Briefly, overnight cultures of the receiver strains were diluted to a 1:50 ratio with glucose-casein hydrolysate-potassium phosphate buffer (GCHE) medium and incubated for 4 hours at 37°C. After incubation, 5 to 10 μg of genomic or plasmid DNA were mixed with 400 μl of competent cells and were further incubated for 2 hours before plating on lysogeny broth (LB) agar containing selective antibiotics. To create double-deletion mutants, PS216, ∆*ytvA*::Km^R^*, sacA*::P*_ytvA_*-*luxABCDE* (Cm^R^) strain was transformed with a temperature-sensitive plasmid with Cre recombinase, pMH66, and transformants were selected on LB (Lennox, Carl Roth, Karlsruhe, Germany) plates supplemented with tetracycline (10 μg ml^−1^) at 37°C. Loss of Km^R^ marker was tested on LB agar medium containing kanamycin (5 μg ml^−1^) at 37°C. Candidates that were not able to grow on kanamycin were further incubated on LB plates at 43°C for 18 hours to lose pMH66. Strains that were unable to grow on LB plates supplemented with tetracycline were considered to have lost pMH66 and antibiotic resistance cassettes. Markerless deletion was confirmed by polymerase chain reaction (PCR) using the primers listed below. Subsequently, the second gene deletion was introduced.

### Growth conditions

For routine growth, bacterial cells were cultured in LB [tryptone (10 g liter^−1^), yeast extract (5 g liter^−1^), and NaCl (5 g liter^−1^)]. For all luminometry experiments shown, bacteria were grown overnight in LB medium after which they were diluted to optical density (OD) of 0.05 and grown in nutrient sporulation medium (NSMP) (*62*) [nutrient broth (8 g l^−1^) (Difco), 1 μM FeCl_3_, 700 μM CaCI_2_, 50 μM MnCl_2_, 1 mM MgCl_2_, and 100 mM potassium phosphate]. Medium was supplemented with 0.1% (w/v) glucose, except when bacterial cultures were entrained for 5 days (Fig. 1), in which case medium was supplemented with 0.05% (w/v) glucose.

### Luminometry experiments

White, 96-well plates (Nunclon Delta, Thermo Fisher Scientific) were used, with each well inoculated with 5 × 10^5^ cells. Plates were sealed with a transparent, evaporation-free cover (Optical Adhesive Covers, Applied Biosystems, Life Technologies). Cultures were exposed to light-darkness cycles as indicated in the text, after which the cultures were released to constant conditions (either constant darkness or light). The temperature was kept constant at 27°C. We measured bioluminescence (Berthold Centro LB960 XS3 or Berthold Mithras LB 940 Multimode Plate Reader) for 1 s each hour. All experiments were carried out in temperature-controlled incubators (MIR-154, Panasonic, Japan or Percival Intellus, Percival, USA). In entrainment experiments, the plates were ejected from the machine between readings for exposure to light.

### Measurement of GFP fluorescence using the microplate reader

Cultures were prepared and grown in NSMP medium supplemented with 0.1% (w/v) glucose as described for the luminometry experiment, with the differences that black plates (Nunclon Delta, Thermo Fisher Scientific) were used to reduce background signal. Cultures were exposed to light-darkness cycles for 3 days, after which the cultures were released to constant darkness conditions. The temperature was kept constant at 27°C. GFP fluorescence was red every hour using a multimode plate reader (Berthold Mithras LB 940 Multimode Plate Reader). All experiments were carried out in temperature-controlled incubators (Percival Intellus, Percival, USA). The plate was ejected from the machine between readings, for exposure to light. Signal from control wells (*n* = 23) with bacteria carrying no fluorescence reporter was subtracted as background.

### Assessment of cell number

Cultures were grown in 96-well plates in NSMP medium supplemented with 0.1% (w/v) glucose as described for the luminometry experiments. Bacteria were exposed to 24 bLD cycles for 3 days, after which cultures were released to constant darkness. Fluence rate in the light phase was 30 μE m^−2^ s^−1^. The temperature was kept constant at 27°C during the all experiment. We determined cell number from day 3 (last day of the entraining cycle) and the first day in constant darkness conditions (free-run) harvesting samples every 4 hour and every 12 hours for the following 36 hours. Samples were sonicated (Diagenode Bioruptor, USA) at low power (130 W) for 15 s, for four cycles, with a 5-s pause between cycles, a protocol that we optimized in our laboratory that allows proper disruption of biofilms without affecting cell viability. Sonicated cells were examined by light microscopy (Leica, Germany) to confirm disruption of biofilm and cell viability. If biofilm was not successfully disrupted, samples were subject to more cycles of sonication until no more biofilm was visible under light microscopy. Following serial dilutions, the sonicated cells were plated on LB agar and grown overnight at 37°C. The number of colony-forming units was counted on the following day.

### RT-qPCR

Bacteria at OD 0.5 were diluted 1:25 in NSMP medium supplemented with 0.1% (w/v) glucose in 100-ml Erlenmeyer flask (20 ml of culture per flask). Cultures were grown shaking at 120 rpm for 48 hours in darkness and then were either exposed to either blue light (30 μE m^−2^ s^−1^) or were kept in darkness as control. Temperature was kept constant at 27°C. Immediately before blue light illumination (time 0) and after 1 and 4 hours of blue light treatment alongside dark control, samples were pelleted and flash-frozen in liquid nitrogen. Pellets were lysed in lysozyme digestion buffer [lysozyme (10 mg/ml), 30 mM Tris-HCl (pH 6.3), and 10 mM EDTA] for RNA extraction with TRIzol and the Direct-zol RNA extraction kit (Zymo Research), followed by cleaning with the GeneJet RNA Cleanup and Concentration micro kit (Thermo Fisher Scientific) according to manufacturer’s protocol. The total RNA concentration was measured using a NanoDrop 1000 spectrophotometer (Peqlab Biotechnologie). Equal amounts of complementary DNA were synthesized using iScript Reverse Transcription Supermix (Bio-Rad) following the manufacturer’s protocol. Real-time quantitative PCR (RT-qPCR) was performed using the SYBR Select Master Mix (Applied Biosystems, Thermo Fisher Scientific) on a ViiA7 real-time PCR system (Applied Biosystems, Thermo Fisher Scientific). The mRNA level of the *ytvA* target gene in each sample was normalized to the amount of 16*S* gene as reference control. The primers used for *ytvA* amplification are as follow: 5′-TCCCTCACGGAAATTACTGC-3′ and 5′-GGCTCAGCTTGAAAATTTGG-3′. The primers used for 16*S* amplification are as follows: 5′-GCTCGTGTCGTGAGATGTTGGGTTA-3′ and 5-GGTTTCGCTGCCCTTTGTTCTGTCC-3′. The *ytvA* mRNA fold changes were determined using the ΔΔ*C*_t_ method including three technical replicates per condition.

### Light settings

Samples were exposed to light using blue or red light-emitting diodes with peak emission at, respectively, 450 or 625 nm (Barthelme, Nürnberg, Germany). The average photon flux density for each experiment is indicated in the text and/or the figure legends. Neutral density filters were used to achieve the desired illumination intensity (Rosco Supergel 398 Neutral Grey or Rosco E-colour E299 1.2 Neutral Density, Lightpower GmbH, Paderborn, DE). Light intensity was measured using a radiometer/photometer (Model IL1400A, International Light Inc., Newburyport, MA, USA). Temperature fluctuations in the incubator due to lights being on or off were less than 0.5°C.

### Data analysis

Data are expressed as mean ± SD. Sample sizes are indicated in figure legends and in table S3. One sample is equals to one well of a microreader plate.

### Analysis of FRP following 5 days of entrainment with LD cycles or in absence of entrainment

For data shown in Fig. 1, data analysis of period was performed using Fast Fourier Transform nonlinear least squares function of the BioDare2 suite (*63*) only for samples that passed the Jonckheere-Terpstra-Kendall (JTK) cycle test for rhythmicity (*P* < 0.05) on nonnormalized, baseline detrended data. The *P* values were corrected using the Benjamini-Hochberg procedure for multiple testing. Periods were considered as circadian when their duration was between 18 and 34 hours.

### Analysis of FRP of the strain PS216 following 3 days of entrainment with LD cycles

Rhythms of LUC activity were analyzed as described above, with the differences that data were linear detrended and that rhythmicity was analyzed using JTK cycle with a statistical cutoff of *P* < 0.01, adjusted for multiple testing using the Benjamini-Hochberg procedure.

All statistical analyses were carried out using Prism 8 software version 8.4.3. Normality was checked using the Shapiro-Wilk test. When samples were normally distributed, means between two groups of values were compared using the two-sample Student’s *t* test with 95% confidence interval and one-way ANOVA was used to compare the means between more than two groups. When the assumption of normality was not met, Mann-Whitney and Kruskal-Wallis tests were performed to compare two or more groups, respectively. For statistical analysis including two factors (time and type of illumination; Fig. 2), a two-way ANOVA followed by Tukey’s multiple comparison test was performed.

### Linear mixed-effects model

To determine the effect of light on period or the first peak after release, we applied a linear mixed-effects modeling approach. For each fluence rate assessed, two datasets coming from two independent experiments (96-well plates) were included in the analysis. The effect “plate” (biological replicate) was included as a random effect intercept in the model. Statistical modeling was performed using R version 4.1.2 and the tidyr, stringr, lme4, mgcv, and dplyr packages therein. The model was as follow

model<−gam(value∼light_group+s(plate,bs='re'),data=light,method=″REML″)

The full reproducible code is provided in data file S1.

### Analysis of center of gravity

The center of gravity was calculated as described in (*64*) using the formulae

$$a=\frac{\sum_{i=1}^{n}y_{1}\cos\frac{2\pi i}{n}}{\sum_{i=1}^{n}y_{1}};\;b=\frac{\sum_{i=1}^{n}y_{1}\sin\frac{2\pi i}{n}}{\sum_{i=1}^{n}y_{1}};\;\frac{b}{a}=\frac{\sum_{i=1}^{n}y_{1}\sin\frac{2\pi i}{n}}{\sum_{i=1}^{n}y_{1}\cos\frac{2\pi i}{n}}$$

The code of the software we developed for calculation is available here: https://github.com/luca-gas/center-of-gravity

### Plotting data

Graphs were generated using either GraphPad Prism software version 8.4.3 [GraphPad Software, La Jolla, CA] or the ggplot2 package in R version 4.1.2 (*65*)], except for double-plot actograms, which were created using ChronoSapiens program version 9 (Chronsulting).

## Supplementary Materials

### This PDF file includes:

Figs. S1 to S6 Tables S1 to S3 Data S1 References

## References

[1] Y. M. Bar-On, R. Phillips, R. Milo, The biomass distribution on Earth. Proc. Natl. Acad. Sci. U.S.A. 115, 6506–6511 (2018). 29784790 10.1073/pnas.1711842115 PMC6016768

[2] M. I. Soriano, B. Roibás, A. B. García, M. Espinosa-Urgel, Evidence of circadian rhythms in non-photosynthetic bacteria? J. Circadian Rhythms 8, 8 (2014). 10.1186/1740-3391-8-8 PMC2949598 20846401

[3] J. K. Paulose, J. M. Wright, A. G. Patel, V. M. Cassone, Human gut bacteria are sensitive to melatonin and express endogenous circadian rhythmicity. PLOS ONE 11, e0146643 (2016). 26751389 10.1371/journal.pone.0146643 PMC4709092

[4] J. K. Paulose, C. V. Cassone, K. B. Graniczkowska, V. M. Cassone, Entrainment of the circadian clock of the enteric bacterium *Klebsiella aerogenes* by temperature cycles. iScience 19, 1202–1213 (2019). 31551197 10.1016/j.isci.2019.09.007 PMC6831877

[5] Z. Eelderink-Chen, J. Bosman, F. Sartor, A. N. Dodd, Á. T. Kovács, M. Merrow, A circadian clock in a nonphotosynthetic prokaryote. Sci. Adv. 7, eabe2086 (2021). 33523996 10.1126/sciadv.abe2086 PMC7793578

[6] J. Aschoff, Exogenous and endogenous components in circadian rhythms. Cold Spring Harb. Symp. Quant. Biol. 25, 11–28 (1960). 13684695 10.1101/sqb.1960.025.01.004

[7] S. S. Branda, Å. Vik, L. Friedman, R. Kolter, Biofilms: The matrix revisited. Trends Microbiol. 13, 20–26 (2005). 15639628 10.1016/j.tim.2004.11.006

[8] R. Gallegos-Monterrosa, E. Mhatre, Á. T. Kovács, Specific *Bacillus subtilis* 168 variants form biofilms on nutrient-rich medium. Microbiology 162, 1922–1932 (2016). 27655338 10.1099/mic.0.000371

[9] D. B. Kearns, R. Losick, Swarming motility in undomesticated *Bacillus subtilis*. Mol. Microbiol. 49, 581–590 (2003). 12864845 10.1046/j.1365-2958.2003.03584.x

[10] P. Stefanic, I. Mandic-Mulec, Social interactions and distribution of *Bacillus subtilis* pherotypes at microscale. J. Bacteriol. 191, 1756–1764 (2009). 19114482 10.1128/JB.01290-08 PMC2648371

[11] H. T. Kiesewalter, C. N. Lozano-Andrade, M. Wibowo, M. L. Strube, G. Maróti, D. Snyder, T. S. Jørgensen, T. O. Larsen, V. S. Cooper, T. Weber, Á. T. Kovács, Genomic and chemical diversity of *Bacillus subtilis* secondary metabolites against plant pathogenic fungi. mSystems 6, e00770–e007720 (2021). 10.1128/mSystems.00770-20 PMC8573961 33622852

[12] J. M. Hurley, A. Dasgupta, J. M. Emerson, X. Zhou, C. S. Ringelberg, N. Knabe, A. M. Lipzen, E. A. Lindquist, C. G. Daum, K. W. Barry, I. V. Grigoriev, K. M. Smith, J. E. Galagan, D. Bell-Pedersen, M. Freitag, C. Cheng, J. J. Loros, J. C. Dunlap, Analysis of clock-regulated genes in *Neurospora* reveals widespread posttranscriptional control of metabolic potential. Proc. Natl. Acad. Sci. U.S.A. 111, 16995–17002 (2014). 25362047 10.1073/pnas.1418963111 PMC4260557

[13] D. K. Welsh, S.-H. Yoo, A. C. Liu, J. S. Takahashi, S. A. Kay, Bioluminescence imaging of individual fibroblasts reveals persistent, independently phased circadian rhythms of clock gene expression. Curr. Biol. 14, 2289–2295 (2004). 15620658 10.1016/j.cub.2004.11.057 PMC3777438

[14] F. E. Belbin, Z. B. Noordally, S. J. Wetherill, K. A. Atkins, K. A. Franklin, A. N. Dodd, Integration of light and circadian signals that regulate chloroplast transcription by a nuclear-encoded sigma factor. New Phytol. 213, 727–738 (2017). 27716936 10.1111/nph.14176 PMC5215360

[15] T. Roenneberg, S. Daan, M. Merrow, The art of entrainment. J. Biol. Rhythms 18, 183–194 (2003). 12828276 10.1177/0748730403018003001

[16] M. Ávila-Pérez, J. B. van der Steen, R. Kort, K. J. Hellingwerf, Red light activates the σ^B^-mediated general stress response of *Bacillus subtilisvia* the energy branch of the upstream signaling cascade. J. Bacteriol. 192, 755–762 (2010). 19948797 10.1128/JB.00826-09 PMC2812468

[17] J. Aschoff, C. von Goetz, Masking of circadian activity rhythms in hamsters by darkness. J. Comp. Physiol. A 162, 559–562 (1988). 3361461 10.1007/BF00612521

[18] H. G. McWatters, R. M. Bastow, A. Hall, A. J. Millar, The *ELF3 zeitnehmer* regulates light signalling to the circadian clock. Nature 408, 716–720 (2000). 11130072 10.1038/35047079

[19] J. Rémi, M. Merrow, T. Roenneberg, A circadian surface of entrainment: Varying T, τ, and photoperiod in *Neurospora crassa*. J. Biol. Rhythms 25, 318–328 (2010). 20876812 10.1177/0748730410379081

[20] Y. Tan, Z. Dragovic, T. Roenneberg, M. Merrow, Entrainment dissociates transcription and translation of a circadian clock gene in *Neurospora*. Curr. Biol. 14, 433–438 (2004). 15028220 10.1016/j.cub.2004.02.035

[21] V. G. Bruce, Environmental entrainment of circadian rhythms. Cold Spring Harb. Symp. Quant. Biol. 25, 29–48 (1960). 10.1101/sqb.1960.025.01.033 13762942

[22] M. Merrow, M. Brunner, T. Roenneberg, Assignment of circadian function for the *Neurospora* clock gene frequency. Nature 399, 584–586 (1999). 10376598 10.1038/21190

[23] T. Roenneberg, Z. Dragovic, M. Merrow, Demasking biological oscillators: Properties and principles of entrainment exemplified by the *Neurospora* circadian clock. Proc. Natl. Acad. Sci. U.S.A. 102, 7742–7747 (2005). 15899977 10.1073/pnas.0501884102 PMC1140435

[24] C. S. Pittendrigh, S. Daan, A Functional analysis of circadian pacemakers in nocturnal rodents. J. Comp. Physiol. A 106, 223–252 (1976).

[25] C. S. Pittendrigh, Circadian rhythms and the circadian organization of living systems. Cold Spring Harb. Symp. Quant. Biol. 25, 159–184 (1960). 13736116 10.1101/sqb.1960.025.01.015

[26] D. Bates, M. Mächler, B. M. Bolker, S. C. Walker, Fitting linear mixed-effects models using lme4. J. Stat. Softw. 67, 1–48 (2015).

[27] M. M. Canal-Corretger, J. Vilaplana, T. Cambras, A. Díez-Noguera, Effect of light on the development of the circadian rhythm of motor activity in the mouse. Chronobiol. Int. 18, 683–696 (2001). 11587090 10.1081/cbi-100106081

[28] C. H. Johnson, J. W. Hastings, Circadian phototransduction: Phase resetting and frequency of the circadian clock of *Gonyaulax* cells in red light. J. Biol. Rhythms 4, 417–437 (1989). 2519604 10.1177/074873048900400403

[29] T. Roenneberg, D. Morse, Two circadian oscillators in one cell. Nature 362, 362–364 (1993). 29634015 10.1038/362362a0

[30] M. L. Pay, D. W. Kim, D. E. Somers, J. K. Kim, M. Foo, Modelling of plant circadian clock for characterizing hypocotyl growth under different light quality conditions. In Silico Plants 4, diac001 (2022). 35369361 10.1093/insilicoplants/diac001 PMC8963510

[31] T. Ohara, H. Fukuda, I. T. Tokuda, An extended mathematical model for reproducing the phase response of *Arabidopsis thaliana* under various light conditions. J. Theor. Biol. 382, 337–344 (2015). 26231414 10.1016/j.jtbi.2015.07.016

[32] T. Woelders, E. J. Wams, M. C. M. Gordijn, D. G. M. Beersma, R. A. Hut, Integration of color and intensity increases time signal stability for the human circadian system when sunlight is obscured by clouds. Sci. Rep. 8, 15214 (2018). 30315193 10.1038/s41598-018-33606-5 PMC6185968

[33] H. G. Nimmo, Entrainment of *Arabidopsis* roots to the light:dark cycle by light piping. Plant Cell Environ. 41, 1742–1748 (2018). 29314066 10.1111/pce.13137

[34] J. T. Woolley, E. W. Stoller, Light penetration and light-induced seed germination in soil. Plant Physiol. 61, 597–600 (1978). 16660344 10.1104/pp.61.4.597 PMC1091925

[35] Y. Ouyang, C. R. Andersson, T. Kondo, S. S. Golden, C. H. Johnson, Resonating circadian clocks enhance fitness in cyanobacteria. Proc. Natl. Acad. Sci. U.S.A. 95, 8660–8664 (1998). 9671734 10.1073/pnas.95.15.8660 PMC21132

[36] A. N. Dodd, N. Salathia, A. Hall, E. Kévei, R. Tóth, F. Nagy, J. M. Hibberd, A. J. Millar, A. A. R. Webb, Plant circadian clocks increase photosynthesis, growth, survival, and competitive advantage. Science 309, 630–633 (2005). 16040710 10.1126/science.1115581

[37] S. Daan, K. Spoelstra, U. Albrecht, I. Schmutz, M. Daan, B. Daan, F. Rienks, I. Poletaeva, G. Dell’Omo, A. Vyssotski, H.-P. Lipp, Lab mice in the field: Unorthodox daily activity and effects of a dysfunctional circadian clock allele. J. Biol. Rhythms 26, 118–129 (2011). 21454292 10.1177/0748730410397645

[38] A. Newman, E. Picot, S. Davies, S. Hilton, I. A. Carré, G. D. Bending, Circadian rhythms in the plant host influence rhythmicity of rhizosphere microbiota. BMC Biol. 20, 235 (2022). 36266698 10.1186/s12915-022-01430-z PMC9585842

[39] M. Ávila-Pérez, K. J. Hellingwerf, R. Kort, Blue light activates the σ^B^-dependent stress response of *Bacillus subtilis* via YtvA. J. Bacteriol. 188, 6411–6414 (2006). 16923909 10.1128/JB.00716-06 PMC1595387

[40] T. A. Gaidenko, T. J. Kim, A. L. Weigel, M. S. Brody, C. W. Price, The blue-light receptor YtvA acts in the environmental stress signaling pathway of *Bacillus subtilis*. J. Bacteriol. 188, 6387–6395 (2006). 16923906 10.1128/JB.00691-06 PMC1595380

[41] S. Akbar, T. A. Gaidenko, C. Min Kang, M. O’Reilly, K. M. Devine, C. W. Price, New family of regulators in the environmental signaling pathway which activates the general stress transcription factor ampt;sigmaf;^B^ of *Bacillus subtilis*. J. Bacteriol. 183, 1329–1338 (2001). 11157946 10.1128/JB.183.4.1329-1338.2001 PMC95007

[42] M. Avila-Pérez, J. Vreede, Y. Tang, O. Bende, A. Losi, W. Gärtner, K. Hellingwerf, *In vivo* mutational analysis of YtvA from *Bacillus subtilis*: Mechanism of light activation of the general stress response. J. Biol. Chem. 284, 24958–24964 (2009). 19581299 10.1074/jbc.M109.033316 PMC2757199

[43] R. S. Edgar, E. W. Green, Y. Zhao, G. Van Ooijen, M. Olmedo, X. Qin, Y. Xu, M. Pan, U. K. Valekunja, K. A. Feeney, E. S. Maywood, M. H. Hastings, N. S. Baliga, M. Merrow, A. J. Millar, C. H. Johnson, C. P. Kyriacou, J. S. O’Neill, A. B. Reddy, Peroxiredoxins are conserved markers of circadian rhythms. Nature 485, 459–464 (2012). 22622569 10.1038/nature11088 PMC3398137

[44] A. G. Albrecht, D. J. A. Netz, M. Miethke, A. J. Pierik, O. Burghaus, F. Peuckert, R. Lill, M. A. Marahiel, SufU is an essential iron-sulfur cluster scaffold protein in *Bacillus subtilis*. J. Bacteriol. 192, 1643–1651 (2010). 20097860 10.1128/JB.01536-09 PMC2832514

[45] Y. A. Oñate, S. J. Vollmer, R. L. Switzer, M. K. Johnson, Spectroscopic characterization of the iron-sulfur cluster in *Bacillus subtilis* glutamine phosphoribosylpyrophosphate amidotransferase. J. Biol. Chem. 264, 18386–18391 (1989). 2553706

[46] M. M. Blahut, E. Sanchez, C. E. Fisher, F. W. Outten, Fe-S cluster biogenesis by the bacterial Suf pathway. Biochim. Biophys. Acta Mol. Cell. Res. 1867, 118829 (2020). 32822728 10.1016/j.bbamcr.2020.118829 PMC7510350

[47] L. Hederstedt, A. Lewin, M. Throne-Holst, Heme A synthase enzyme functions dissected by mutagenesis of *Bacillus subtilis* CtaA. J. Bacteriol. 187, 8361–8369 (2005). 16321940 10.1128/JB.187.24.8361-8369.2005 PMC1317025

[48] N. B. Ivleva, M. R. Bramlett, P. A. Lindahl, S. S. Golden, LdpA: A component of the circadian clock senses redox state of the cell. EMBO J. 24, 1202–1210 (2005). 15775978 10.1038/sj.emboj.7600606 PMC556408

[49] M. J. Rust, S. S. Golden, E. K. O’Shea, Light-driven changes in energy metabolism directly entrain the cyanobacterial circadian oscillator. Science 331, 220–223 (2011). 21233390 10.1126/science.1197243 PMC3309039

[50] D. B. Pedrolli, C. Kühm, D. C. Sévin, M. P. Vockenhuber, U. Sauer, B. Suess, M. Mack, A dual control mechanism synchronizes riboflavin and sulphur metabolism in *Bacillus subtilis*. Proc. Natl. Acad. Sci. U.S.A. 112, 14054–14059 (2015). 26494285 10.1073/pnas.1515024112 PMC4653141

[51] A. B. James, J. A. Monrea, G. A. Nimmo, C. L. Kelly, P. Herzyk, G. I. Jenkins, H. G. Nimmo, The circadian clock in *Arabidopsis* roots is a simplified slave version of the clock in shoots. Science 322, 1832–1835 (2008). 19095940 10.1126/science.1161403

[52] M. Endo, H. Shimizu, M. A. Nohales, T. Araki, S. A. Kay, Tissue-specific clocks in *Arabidopsis* show asymmetric coupling. Nature 515, 419–422 (2014). 25363766 10.1038/nature13919 PMC4270698

[53] C. Schmal, E. D. Herzog, H. Herzel, Measuring relative coupling strength in circadian systems. J. Biol. Rhythms 33, 84–98 (2018). 29219034 10.1177/0748730417740467 PMC6344889

[54] C. J. Guenthner, M. E. Luitje, L. A. Pyle, P. C. Molyneux, J. K. Yu, A. S. Li, T. L. Leise, M. E. Harrington, Circadian rhythms of PER2::LUC in individual primary mouse hepatocytes and cultures. PLOS ONE 9, e87573 (2014). 24498336 10.1371/journal.pone.0087573 PMC3911982

[55] Y. Hirata, R. Enoki, K. Kuribayashi-Shigetomi, Y. Oda, S. Honma, K.-I. Honma, Circadian rhythms in *Per1*, PER2 and Ca^2+^ of a solitary SCN neuron cultured on a microisland. Sci. Rep. 9, 18271 (2019). 31797953 10.1038/s41598-019-54654-5 PMC6892917

[56] A. Frank, C. C. Matiolli, A. J. C. Viana, T. J. Hearn, J. Kusakina, F. E. Belbin, D. Wells Newman, A. Yochikawa, D. L. Cano-Ramirez, A. Chembath, K. Cragg-Barber, M. J. Haydon, C. T. Hotta, M. Vincentz, A. A. R. Webb, A. N. Dodd, Circadian entrainment in *Arabidopsis* by the sugar-responsive transcription factor bZIP63. Curr. Biol. 28, 2597–2606.e6 (2018). 30078562 10.1016/j.cub.2018.05.092 PMC6108399

[57] E. S. Schernhammer, F. Laden, F. E. Speizer, W. C. Willett, D. J. Hunter, I. Kawachi, G. A. Colditz, Rotating night shifts and risk of breast cancer in women participating in the Nurses’ Health Study. J. Natl. Cancer Inst. 93, 1563–1568 (2001). 11604480 10.1093/jnci/93.20.1563

[58] E. S. Schernhammer, F. Laden, F. E. Speizer, W. C. Willett, D. J. Hunter, I. Kawachi, C. S. Fuchs, G. A. Colditz, Night-shift work and risk of colorectal cancer in the Nurses’ Health Study. J. Natl. Cancer Inst. 95, 825–828 (2003). 12783938 10.1093/jnci/95.11.825

[59] T. Roenneberg, M. Merrow, Molecular circadian oscillators: An alternative hypothesis. J. Biol. Rhythms 13, 167–179 (1998). 9554578 10.1177/074873098129000011

[60] D. W. Kim, C. Chang, X. Chen, A. C. Doran, F. Gaudreault, T. Wager, G. J. DeMarco, J. K. Kim, Systems approach reveals photosensitivity and PER 2 level as determinants of clock-modulator efficacy. Mol. Syst. Biol. 15, e8838 (2019). 31353796 10.15252/msb.20198838 PMC6613017

[61] J. K. Kim, D. B. Forger, M. Marconi, D. Wood, A. Doran, T. Wager, C. Chang, K. M. Walton, Modeling and validating chronic pharmacological manipulation of circadian rhythms. CPT Pharmacometrics Syst. Pharmacol. 2, e57 (2013). 23863866 10.1038/psp.2013.34 PMC3734602

[62] P. Fortnagel, E. Freese, Analysis of sporulation mutants. II. Mutants blocked in the citric acid cycle. J. Bacteriol. 95, 1431–1438 (1968). 4967197 10.1128/jb.95.4.1431-1438.1968 PMC315104

[63] T. Zielinski, A. M. Moore, E. Troup, K. J. Halliday, A. J. Millar, Strengths and limitations of period estimation methods for circadian data. PLOS ONE 9, e96462 (2014). 24809473 10.1371/journal.pone.0096462 PMC4014635

[64] A. Díez-Noguera, Methods for serial analysis of long time series in the study of biological rhythms. J. Circadian Rhythms 11, 7 (2014). 10.1186/1740-3391-11-7 PMC3723718 23867052

[65] H. Wickham H, *ggplot2: Elegant Graphics for Data Analysis*. (Springer-Verlag New York, 2016).

[66] B.-M. Koo, G. Kritikos, J. D. Farelli, H. Todor, K. Tong, H. Kimsey, I. Wapinski, M. Galardini, A. Cabal, J. M. Peters, A.-B. Hachmann, D. Z. Rudner, K. N. Allen, A. Typas, C. A. Gross, Construction and analysis of two genome-scale deletion libraries for *Bacillus subtilis*. Cell Syst. 4, 291–305.e7 (2017). 28189581 10.1016/j.cels.2016.12.013 PMC5400513

[67] I. Seccareccia, Á. T. Kovács, R. Gallegos-Monterrosa, M. Nett, Unraveling the predator-prey relationship of *Cupriavidus necator* and *Bacillus subtilis*. Microbiol. Res. 192, 231–238 (2016). 27664741 10.1016/j.micres.2016.07.007

[68] Á. T. Kovács, M. van Hartskamp, O. P. Kuipers, R. van Kranenburg, Genetic tool development for a new host for biotechnology, the thermotolerant bacterium *Bacillus coagulans*. Appl. Environ. Microbiol. 76, 4085–4088 (2010). 20400555 10.1128/AEM.03060-09 PMC2893497

[69] J. Husse, A. Leliavski, A. H. Tsang, H. Oster, G. Eichele, The light-dark cycle controls peripheral rhythmicity in mice with a genetically ablated suprachiasmatic nucleus clock. FASEB J. 28, 4950–4960 (2014). 25063847 10.1096/fj.14-256594

[70] S. J. Aton, G. D. Block, H. Tei, S. Yamazaki, E. D. Herzog, Plasticity of circadian behavior and the suprachiasmatic nucleus following exposure to non-24-hour light cycles. J. Biol. Rhythms 19, 198–207 (2004). 15155006 10.1177/0748730404264156

[71] P. C. Molyneux, M. K. Dahlgren, M. E. Harrington, Circadian entrainment aftereffects in suprachiasmatic nuclei and peripheral tissues in vitro. Brain Res. 1228, 127–134 (2008). 18598681 10.1016/j.brainres.2008.05.091

[72] N. Mrosovsky, Aschoff’s rule in retinally degenerate mice. J. Comp. Physiol. A Neuroethol. Sens. Neural Behav. Physiol. 189, 75–78 (2003). 12548433 10.1007/s00359-002-0381-z

[73] S. Steinlechner, B. Jacobmeier, F. Scherbarth, H. Dernbach, F. Kruse, U. Albrecht, Robust circadian rhythmicity of *Per1* and *Per2* mutant mice in constant light, and dynamics of *Per1* and *Per2* gene expression under long and short photoperiods. J. Biol. Rhythms 17, 202–209 (2002). 12054191 10.1177/074873040201700303

[74] B. Thines, F. G. Harmon, Ambient temperature response establishes ELF3 as a required component of the core *Arabidopsis* circadian clock. Proc. Natl. Acad. Sci. U.S.A. 107, 3257–3262 (2010). 20133619 10.1073/pnas.0911006107 PMC2840299

[75] E. Kolmos, E. Herrero, N. Bujdoso, A. J. Millar, R. Tóth, P. Gyula, F. Nagy, S. J. Davis, A reduced-function allele reveals that EARLY FLOWERING3 repressive action on the circadian clock is modulated by phytochrome signals in *Arabidopsis*. Plant Cell 23, 3230–3246 (2011). 21908721 10.1105/tpc.111.088195 PMC3203447

[76] A. N. Dodd, N. Dalchau, M. J. Gardner, S. J. Baek, A. A. R. Webb, The circadian clock has transient plasticity of period and is required for timing of nocturnal processes in *Arabidopsis*. New Phytol. 201, 168–179 (2014). 24102325 10.1111/nph.12489

[77] D. E. Somers, P. F. Devlin, S. A. Kay, Phytochromes and cryptochromes in the entrainment of the *Arabidopsis* circadian clock. Science 282, 1488–1490 (1998). 9822379 10.1126/science.282.5393.1488

[78] Y. He, Y. Yu, X. Wang, Y. Qin, C. Su, L. Wang, Aschoff’s rule on circadian rhythms orchestrated by blue light sensor CRY2 and clock component PRR9. Nat. Commun. 13, 5869 (2022). 36198686 10.1038/s41467-022-33568-3 PMC9535003

[79] J. Diegmann, A. Stück, C. Madeti, T. Roenneberg, Entrainment elicits period aftereffects in *Neurospora crassa*. Chronobiol. Int. 27, 1335–1347 (2010). 20795879 10.3109/07420528.2010.504316

[80] K. Schneider, S. Perrino, K. Oelhafen, S. Li, A. Zatsepin, P. Lakin-Thomas, S. Brody, Rhythmic conidiation in constant light in *Vivid* mutants of *Neurospora crassa*. Genetics 181, 917–931 (2009). 19139144 10.1534/genetics.108.097808 PMC2651064

[81] T. Roenneberg, G. N. Colfax, J. W. Hastings, A circadian rhythm of population behavior in *Gonyaulax polyedra*. J. Biol. Rhythms 4, 201–216 (1989). 2519589

[82] T. Roenneberg, J. W. Hastings, Are the effects of light on phase and period of the *Gonyaulax* clock mediated by different pathways? Photochem. Photobiol. 53, 525–533 (1991). 1857747 10.1111/j.1751-1097.1991.tb03665.x

[83] T. Roenneberg, J. W. Hastings, Two photoreceptors control the circadian clock of a unicellular alga. Naturwissenschaften 75, 206–207 (1988). 3398926 10.1007/BF00735584

[84] J. Toepel, J. E. McDermott, T. C. Summerfield, L. A. Sherman, Transcriptional analysis of the unicellular, diazotrophic cyanobacterium *Cyanothece* sp. ATCC 51142 grown under short day/night cycles. J. Phycol. 45, 610–620 (2009). 27034037 10.1111/j.1529-8817.2009.00674.x

[85] T. Yoshida, Y. Murayama, H. Ito, H. Kageyama, T. Kondo, Nonparametric entrainment of the in vitro circadian phosphorylation rhythm of cyanobacterial KaiC by temperature cycle. Proc. Natl. Acad. Sci. U.S.A. 106, 1648–1653 (2009). 19164549 10.1073/pnas.0806741106 PMC2635835

[86] S. R. Mackey, J. L. Ditty, E. M. Clerico, S. S. Golden, Detection of rhythmic bioluminescence from luciferase reporters in cyanobacteria. Methods Mol. Biol. 362, 115–129 (2007). 17417005 10.1007/978-1-59745-257-1_8

[87] M. Katayama, T. Kondo, J. Xiong, S. S. Golden, *ldpA* encodes an iron-sulfur protein involved in light-dependent modulation of the circadian period in the cyanobacterium *Synechococcus elongatus* PCC 7942. J. Bacteriol. 185, 1415–1422 (2003). 12562813 10.1128/JB.185.4.1415-1422.2003 PMC142860

[88] R Core Team, R: A language and environment for statistical computing. (R Foundation for Statistical Computing, 2021). https://www.R-project.org/.

