# Supplementary material for: The circadian clock of the bacterium B. subtilis evokes properties of complex, multicellular circadian systems
Source: Sci Adv. 2023 Aug 4;9(31):eadh1308. doi: 10.1126/sciadv.adh1308 (PMC10403212; doi:10.1126/sciadv.adh1308)
Supplement: Supplementary file 1 — Figs. S1 to S6 Tables S1 to S3 Data S1 References [file sciadv.adh1308_sm.pdf]

Science Advances
AAAS

Supplementary Materials for

The circadian clock of the bacterium *B. subtilis* evokes properties of complex, multicellular circadian systems

Francesca Sartor *et al.*

Corresponding author: Francesca Sartor, francesca.sartor@med.uni-muenchen.de;
Martha Merrow, merrow@lmu.de

Sci. Adv. **9**, eadh1308 (2023)
DOI: 10.1126/sciadv.adh1308

This PDF file includes:

Figs. S1 to S6
Tables S1 to S3
Data S1
References

Fig. S1.

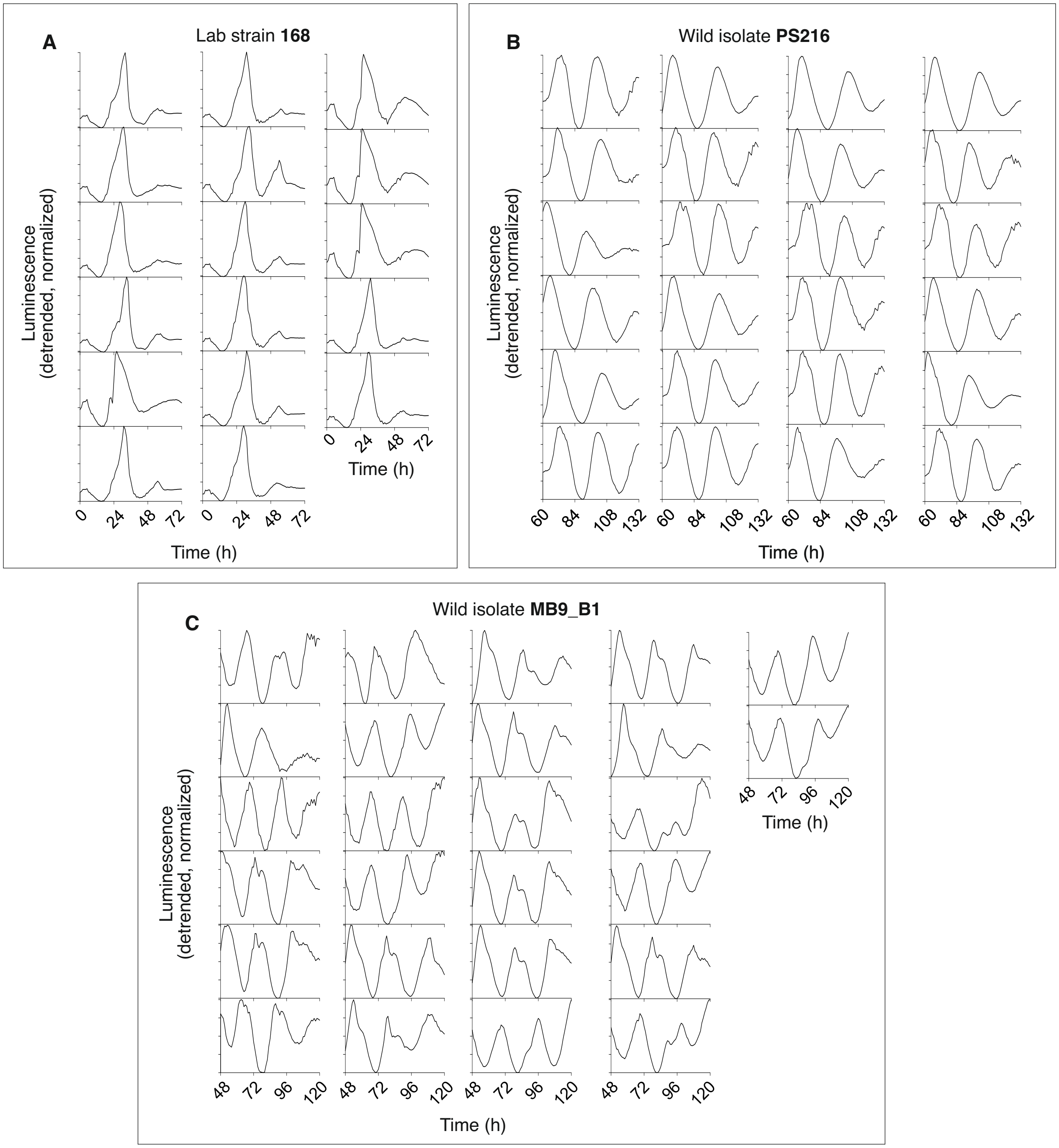

Fig. S1: **Single traces of 168, PS216, and MB9_B1 cultures exhibiting oscillations in P$_{ytvA}$-*lux* reporter from cultures grown in constant conditions from the beginning of the experiment.** The strains P$_{ytvA}$-*lux* 168 (A) (n=17), P$_{ytvA}$-*lux* PS216 (B) (n=24) and P$_{ytvA}$-*lux* MB9_B1 (C) (n=26) were incubated in DD for 7 days. Shown is a 72h time window when samples were rhythmic. Baseline-detrended, normalized luminescence is shown. Bacteria were grown in constant darkness at a constant temperature of 27 °C.

Fig. S2.

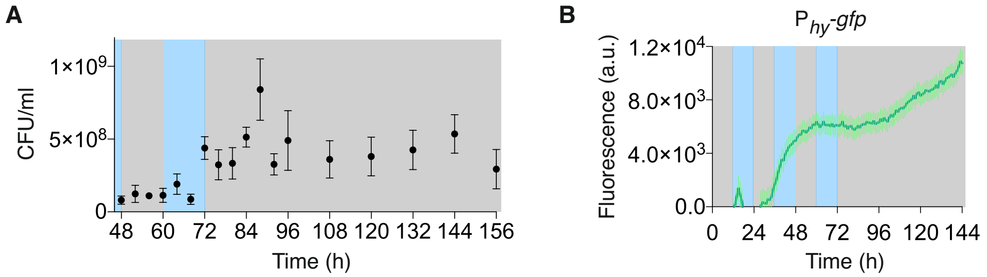

Fig. S2: Cell growth during light entrainment and free-run. A) Cell count in sonicated biofilm. The graph shows the cell number (quantified in CFUs per ml) every 4 hours during the last day of entrainment and the first day in free-run, and every 12 h afterwards until h156. Data represent averages of 3 technical replicates with SEM. **B)** GFP fluorescence shown for the TB269 strain (PS216 with P_{hy}-*gfp*) constitutively expressing GFP (n=24). Shown are mean traces with SEM. A, B) Cultures were exposed to 3 days of entrainment with bLD cycles and subsequent release in constant darkness. Blue and grey areas indicate, respectively, the blue light and the dark phases of the zeitgeber cycles. Bacteria were grown at a constant temperature of 27 °C.

Fig. S3.

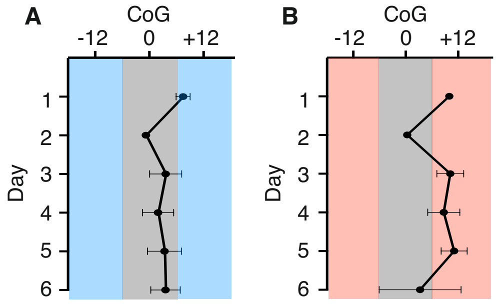

Fig. S3: Center of Gravity (CoG) of P_{ytvA}-*lux* in blue light (n=18) (A) or red light (n=27) (B) LD cycles. The CoG was calculated in reference to the middle of the dark phase. Mean and SD are plotted. Red, blue and grey areas indicate the red light, blue light and the dark phases, respectively. CoG calculated between days 3 and 5 in reference to midnight for samples under bLD cycles is 2.97 ± 0.90 h and for samples under rLD cycles is 9.99 ± 1.20 h.

Fig. S4.

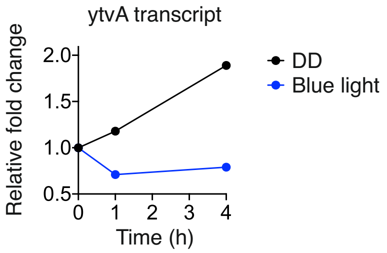

Fig. S4: **Downregulation of *ytvA* transcript levels following exposure to blue light.** Relative abundance of the *ytvA* transcript normalized to the t=0 time point (48h of growth). Blue light fluence rate was 30 µE m^{-2} s^{-1}. The relative abundance of the *ytvA* transcript was determined by RT-qPCR. Values were normalized to the mRNA of the 16S gene. The values shown represent mean relative fold change of *ytvA* transcript levels from three technical replicates.

Fig. S5.

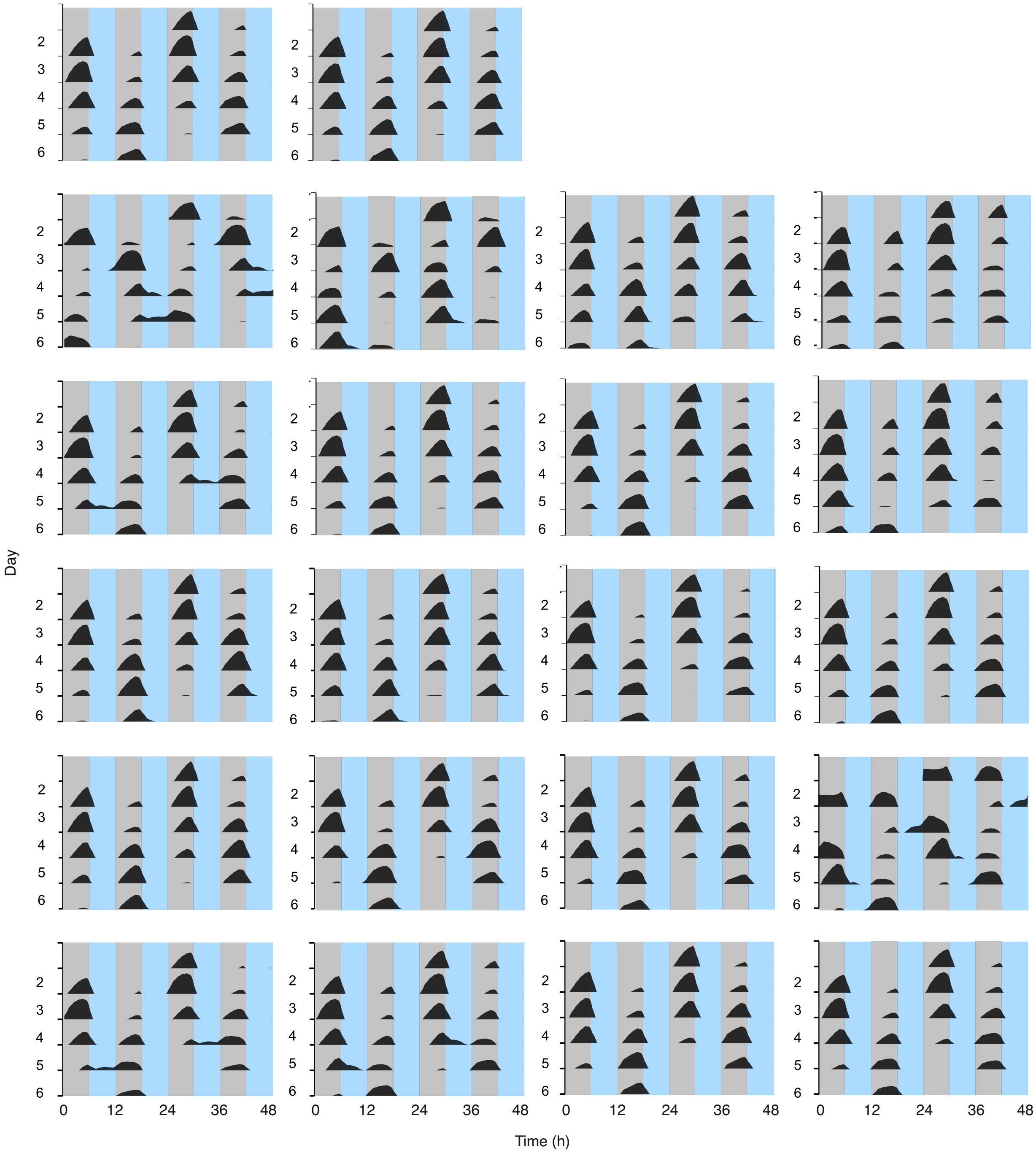

Fig. S5: **Frequency demultiplication occurs in 12h bLD cycles.** PS216 cultures (n=22) expressing the P_{ytvA}-*lux* reporter were incubated for 6 days in 12h bLD cycles (6h darkness/6h light at 30 µE m^{-2} s^{-1}). Gray and blue areas denote, respectively, the dark and the light phases. Temperature was kept constant at 27 °C. Profiles correspond to smoothed, detrended bioluminescence traces of the P_{ytvA}-*lux*. To facilitate visualization, data are double plotted and only positive values are shown.

Fig. S6.

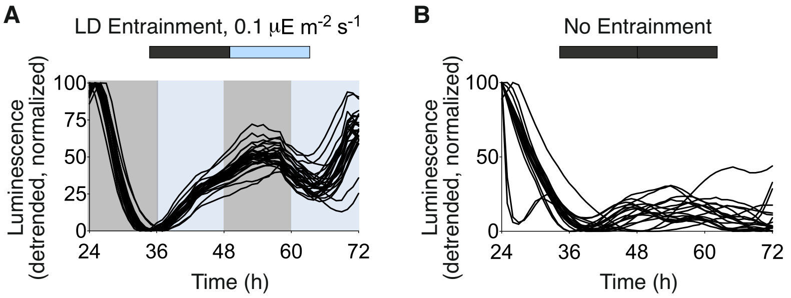

Fig. S6: Comparison of the bioluminescence traces under low intensity bLD cycles relative to DD. A) The strain P_{ytvA}-*lux* PS216 was incubated in bLD cycles for 3 days (12h D/12h L, 0.1 μE m^{-2} s^{-1}) (n=30). P_{ytvA}-*lux* reporter gene expression in the PS216 strain under 12h/12h bLD cycles is shown from 24 to 72 h or incubation. Blue and grey areas indicate the blue light and the dark phases, respectively. Reporter activity was further recorded in control cultures which never experienced zeitgeber cycles (B) (n=18). Temperature was kept constant at 27 °C.

Table S1: Examples of frequency demultiplication, after-effects and Aschoff´s rule in circadian systems across different kingdoms of life.

**Kingdom of life; Species**	**Frequency demultiplication** (Entrainment of circadian oscillator to harmonics of 24h)	**After-effects** (Changes in the free-running period depending on zeitgeber history)	**Aschoff's rule** (FRP in DD or LL changes as a function of irradiance)
Animal; Mouse	Bruce (21) showed that mice (*Peromyscus*) have a single bout of activity per 24h when exposed to LD cycles that are short but a harmonic of 24h (e.g., 8h).<br><br>Entrainment to LD cycles was explored in control and clock mutant mice (69), where *Bmal1* was deleted either in all cells or only in the suprachiasmatic nucleus (SCN). The locomotor activity was monitored when mice were placed under 4h:4h LD cycles. The control mice exhibited frequency demultiplication, while the clock mutants did not, becoming active / inactive each 8h (Figs. 3A and 3B in (69)).	Mice were exposed to an LD cycle length (T) of 20h or 28h (70). The free running period (FRP) of locomotor activity following release to constant conditions was significantly shorter after T20 cycles than after T28. The after-effect was transferred to pups that were born in DD from mothers previously subjected to T20h or T28h. Furthermore, after-effects were observed *in vitro* in the expression of the *Per1-luc* promotor reporter in isolated SCN tissue.<br><br>Mice were exposed to T21h, T26h or T28h LD cycles (71). As in (70), after-effects in FRP were observed for both locomotor activity and *in vitro,* here for PER2::LUC bioluminescence. After-effects *in vitro* were observed for the SCN but not in peripheral tissues (spleen, esophagus, lung and thymus).	FRP of locomotor activity lengthened with increasing levels of light (72). This was observed also in retinally degenerate mice, indicating that rod and cone photoreception is not necessary for Aschoff´s rule.<br><br>A remarkable demonstration of Aschoff's Rule in wild type and clock mutant mice (73). *Per1* mutants generally follow Aschoff's Rule but stay rhythmic (for activity and temperature rhythms) at high light levels. *Per2* mutants defy Aschoff's Rule, showing a shorter FRP in increasing levels of constant light (Fig. 1 in (73).
Plant; *Arabidopsis thaliana*	*TOC1::LUC* expression was monitored in seedlings under T12h temperature cycles (cycles of 6h at 22 °C and 6h at 18 °C (74)). The plants have a major peak of reporter expression every 24h. The *ELF3* gene was	Seedlings were exposed to LD cycles of different duration (T20h, T24h, T28h) (76). Differences in the FRP of bioluminescence reporter constructs were observed after release to constant light.<br>The changes in FRP following entrainment to	The FRP of *CAB2::LUC* shortens with increasing intensity of red light (77). Somers and colleagues tested this response in photoreceptor mutants. They identified phytochrome B and phytochrome A as mediating the response to, respectively, high-

**Kingdom of life; Species**	**Frequency demultiplication** (Entrainment of circadian oscillator to harmonics of 24h)	**After-effects** (Changes in the free-running period depending on zeitgeber history)	**Aschoff's rule** (FRP in DD or LL changes as a function of irradiance)
	implicated in this phenotype, as the *elf3-1* mutant exhibited a driven response (Fig. 2 in (74)).<br><br>*CCR2::LUC* expression was monitored in seedlings under T12h LD cycles (6h light / 6h dark) (75)). Frequency demultiplication occurred in wild-type and *elf3-12* mutant strains. As in (74), frequency demultiplication was impaired in *elf3-1* mutants (Supplemental Fig. 6 in (75)).	different T-cycles decayed (Figs 5 and 6 in (76)).	and low-intensity red light (Fig. 1 in (77)).<br><br>The FRP of *CCA1::LUC* shortens with increasing light intensity under constant red or blue light ((78), Fig. 2b). The authors found that blue light triggers the turnover of the blue light photoreceptor CRY2, in a manner that is proportional to light intensity.
Fungi; *Neurospora crassa*	Frequency demultiplication occurs in 12h temperature cycles of different amplitudes ((22), Fig. 1).	The FRP becomes shorter in samples released from incubation in longer LD T-cycles (79). Clock mutant strains showed the opposite effect (longer FRP corresponding to longer T-cycles) (Fig. 3 in (79)).	Constant light above moonlight levels leads to arrhythmicity in conidiation (22). However, in strains carrying a mutation in the gene *vvd*, conidiation remains rhythmic in LL, with a progressively shorter FRP in increasing intensity of LL (white light) (80), Fig. 3).
SAR (alveolata); *Lingulodinium polyedrum* (formerly known as *Gonyaulax polyedra*)	Swarming rhythm entrains to 6:6 cycles of bright white light / red light with a period of 24h ((81), Fig. 5).	*L. polyedra* free runs in constant blue light (82). A 4h pulse of red light lengthens the FRP of the bioluminescence glow rhythm (Fig. 6 in (82)).	The FRP of the bioluminescent glow rhythm of *L. polyedra* shortens under constant blue or white light, as the light intensity increases (83). Constant red or yellow light has the opposite effect (Fig. 2 in (83)).
Bacteria; cyanobacteria	Nitrogen fixation occurs with a 24h rhythm in *Cyanothece* sp. exposed to 6h:6h LD cycles (Fig. 1A in (84)). Microarray analysis revealed some genes in 6h:6h LD cycles exhibited a driven	To our knowledge, no studies have been performed to explore after-effects in cyanobacteria to date.	The FRP of a bioluminescent reporter (P*psbAI::luxAB*) exhibits a shorter FRP in high light than in low light in *Synechococcus elongatus* (Fig. 4 in (86)).

**Kingdom of life; Species**	**Frequency demultiplication** (Entrainment of circadian oscillator to harmonics of 24h)	**After-effects** (Changes in the free-running period depending on zeitgeber history)	**Aschoff's rule** (FRP in DD or LL changes as a function of irradiance)
	response, while others - including main metabolic genes involved in photosynthesis, respiration, nitrogen fixation, central carbohydrate metabolism and peroxiredoxin - exhibited frequency demultiplication (Fig. 2 and Table 1 in (84)).<br><br>KaiC phosphorylation oscillates with a *ca.* 24h period in *Synechococcus elongatus* PCC7942 exposed to 8:8h cycles of temperature (45 °C/30 °C) in LL (Fig. 5B in (85)).		The FRP of a bioluminescent reporter (P*purF::luxAB*) decreases with increasing white light intensity in *Synechococcus elongatus* (Fig. 5A in (87)).
Bacteria; *Bacillus subtilis*	The expression of a reporter gene P_{ytvA}-*lux* displays frequency demultiplication in T12h symmetrical cycles of blue light/darkness (This study, Fig. 3).	FRP of the P_{ytvA}-*lux* reporter changes systematically when cultures are released to DD from bLD zeitgeber cycles of different amplitudes. The FRP becomes progressively shorter as cultures are entrained to bLD cycles using higher fluence rate (This study, Fig. 4).	*B. subtilis* is rhythmic in both DD and LL; the FRP of the P_{ytvA}-*lux* reporter becomes progressively longer as fluence rate increases (This study, Fig. 5).

Table S2: Statistical comparisons for the effect of light intensity on circadian parameters.

Experiment	Statistical comparison	Reporter strain	Reference to figure in main text
Fluence titration in the entraining LD cycle (after-effects)	FRP in DD following entrainment in bLD cycles of different strength	0 vs 60 µE m^{-2} s^{-1}, $p<0.0001$;<br>0 vs 30 µE m^{-2} s^{-1}, $p<0.0001$;<br>0 vs 3 µE m^{-2} s^{-1}, $p<0.01$;<br>0 vs 0.1 µE m^{-2} s^{-1}, $p<0.05$;<br>0.1 vs 60 µE m^{-2} s^{-1}, $p<0.0001$;<br>0.1 vs 30 µE m^{-2} s^{-1}, $p<0.05$;<br>other comparisons are ns, $p>0.05$.<br>Kruskal-Wallis test followed by Dunn´s multiple comparisons test	Fig. 4L
Release in DD vs LL (Aschoff´s rule)	Estimated FRP lengthening in LL compared to DD	2.97 h (95% CI 0.93-3.18, $p<0.01$) for 30 µE m^{-2} s^{-1} vs DD;<br>2.80 h (95% CI 0.93-3.02, $p<0.01$) for 15 µE $m^{-2}s^{-1}$ vs DD;<br>2.02 h (95% CI 0.94-2.15, $p<0.5$) for 1.5 µE $m^{-2}s^{-1}$ vs DD;<br>linear mixed model (see also Supp. File 2)	Fig. 5E
	FRP in DD and LL, pairwise comparisons	0 vs 30 µE m^{-2} s^{-1}, $p<0.0001$;<br>0 vs 15 µE m^{-2} s^{-1}, $p<0.0001$;<br>0 vs 1.5 µE m^{-2} s^{-1}, $p<0.0001$;<br>30 vs 15 µE m^{-2} s^{-1}, ns, $p>0.05$;<br>30 vs 1.5 µE m^{-2} s^{-1}, $p<0.05$;<br>15 vs 1.5 µE m^{-2} s^{-1}, $p<0.05$.<br>Pairwise comparisons with Bonferroni correction (see also Suppl. File 2)	
	Estimated phase delay after release to LL compared to DD	13.26 h (95% CI 1.84-7.21, $p<0.0001$) for 30 µE m^{-2} s^{-1} vs DD,<br>7.45 h (95% CI 1.82-4.10, $p<0.0001$) for 15 µE m^{-2} s^{-1} vs DD;<br>4.13 h (95% CI 1.87-2.21, $p<0.05$) for 1.5 µE m^{-2} s^{-1} vs DD;<br>linear mixed model (see also Supp. File 2)	Fig. 5F
	Phase after release to DD and LL conditions, pairwise comparisons	All pairwise comparisons with Bonferroni correction were significant ($p<0.001$) (see also Suppl. File 2)	

Table S3: Summary of entrainment conditions and sample sizes. Number of samples (N Rhythmic = numbers of samples exhibiting circadian rhythmicity under free-running conditions, Total N = total number of samples per condition; one sample = one well of a 96-well plate), and period length. Only rhythmic samples are shown in the figures and were included in the analysis for period calculation.

**Experiment**	**Reporter strain**	**LD cycle -> Release**	**Medium**	**N**	**N rhythmic sample/tot N in the exp. (%), mean FRP of the rhythmic samples ± SD**	**Reference to figure in main text**
Different strains, 5 days bLD then release in DD	168, P_{ytvA}-*lux*	30 µE m^{-2} s^{-1} -> DD	NSMP w 0.05% glucose	22	10/22 (45.45%), 30.20 ± 2.33 h	1A-E
	PS216, P_{ytvA}-*lux*			11	8/11 (72.73%), 30.94 ± 2.19 h	
	MB9_B1, P_{ytvA}-*lux*			26	11/26 (42.31%), 29.42 ± 2.52 h	
Different strains, constant DD	168, P_{ytvA}-*lux*	DD, No entrainment	NSMP w 0.05% glucose	32	17/32 (53.13%), 31.11 ± 3.01 h	1F-K, Fig. S1
	PS216, P_{ytvA}-*lux*			32	24/32 (75%), 30.93 ± 1.75 h	
	MB9_B1, P_{ytvA}-*lux*			31	26/31 (83.87%), 25.98 ± 1.82 h	
Entrainment with blue light	PS216, P_{ytvA}-*lux*	15 µE m^{-2} s^{-1}, No release	NSMP w 0.1% glucose	18	N/A	2B, 2C, Fig. S3
Entrainment with red light				27		
T12, bLD cycles	PS216, P_{ytvA}-*lux*	30 µE m^{-2} s^{-1}, No release	NSMP w 0.1% glucose	22	N/A	Fig. 3, Fig. S5
Light fluency titration of the entraining bLD cycle (after-effects)	PS216, P_{ytvA}-*lux*	0 -> DD	NSMP w 0.1% glucose	38	18/38 (47.36%), 29.60 ± 1.32 h	Fig. 4
		100 nE m^{-2} s^{-1} -> DD		31	30/31 (96.77%), 27.45 ± 1.89 h	
		3 µE m^{-2} s^{-1} -> DD		11	11/11 (100%), 26.35 ± 1.82 h	
		30 µE m^{-2} s^{-1} -> DD		18	17/18 (94.44%), 25.36 ± 1.65 h	
		60 µE m^{-2} s^{-1} -> DD		14	14/14 (100%), 24.07 ± 0.68 h	

**Experiment**	**Reporter strain**	**LD cycle -> Release**	**Medium**	**N**	**N rhythmic sample/tot N in the exp. (%), mean FRP of the rhythmic samples ± SD**	**Reference to figure in main text**
Release in DD vs LL (Aschoff´s rule)	PS216, P_{ytvA}-*lux*	30 μE m^{-2} s^{-1} -> DD	NSMP w 0.1% glucose	Exp. 1: 11 Exp. 2: 14	11/11 (100%), 24.68 ± 0.74 h 14/14 (100%), 27.10 ± 1.19 h	Fig. 5
		30 μE m^{-2} s^{-1} -> 1.5 μE m^{-2} s^{-1}		Exp. 1: 25 Exp. 2: 10	22/25 (88%), 27.97 ± 0.85 h 8/10 (80%), 27.88 ± 1.19 h	
		30 μE m^{-2} s^{-1} -> 15 μE m^{-2} s^{-1}		Exp. 1: 38 Exp. 2: 30	31/38 (81.58%), 28.22 ± 0.90 h 28/30 (93.33%), 29.10 ± 0.85 h	
		30 μE m^{-2} s^{-1} -> 30 μE m^{-2} s^{-1}		Exp. 1: 30 Exp. 2: 19	30/30 (100%), 28.62 ± 1.26 h 14/19 (73.68%), 29.15 ± 1.23 h	
Monitoring growth	P_{hy}-*gfp*	30 μE m^{-2} s^{-1} -> DD	NSMP w 0.1% glucose	24	N/A	Fig. S2

Data S1: Code for linear mixed modelling

The following code was used to generate the linear mixed modelling using R version 4.1.2 (88) and the tidyr, stringr, lme4, mgcv and dplyr packages.

```
### loading csv file
light <- read.csv("Light_Francesca.csv")

### get first overview of data
head(light)
str(light)
summary(light)

### transform data from short format to long format
light <- light %>%
  gather(plate) %>%
  na.omit() %>%
  mutate(light_group = str_sub(plate, 1, nchar(plate) - 1),
         plate = as.factor(plate))

### model fit
# fit liner mixed model with random intercept s(plate, bs = 're')
model <- gam(value ~ light_group + s(plate, bs = 're'), data = light, method = "REML")

model_lm <- lm(value ~ light_group, light) # model used for anova to check necessity of the random intercept
anova(model, model_lm) # check necessity of random intercept -> random intercept is significant

summary(model) # coefficients

### diagnostic informations
par(mfrow = c(2, 2)) # combine multiple plots (layout)
gam.check(model)
# left upper corner & left bottom corner: qq-plot check normality of the residuals
# right upper corner: identical to residuals vs. fitted
# right bottom corner: Responses vs. predicted values (fitted)

### pairwise t-test
pairwise.t.test(light$value, light$light_group, p.adjust.method = "bonferroni")
```

Results for the effect of light fluence on period:
value ~ light_group + s(plate, bs = "re")
Parametric coefficients:

	Estimate	Std. Error	t value	Pr(>|t|)	
(Intercept)	25.9061	0.6659	38.904	$< 2e^{-16}$	***

light_groupHighLight	2.9694	0.9335	3.181	0.00178 **
light_groupLowlight	2.0240	0.9424	2.148	0.03333 *
light_groupMediumLight	2.8030	0.9284	3.019	0.00298 **

Signif. codes: 0 '***' 0.001 '**' 0.01 '*' 0.05 '.' 0.1 ' ' 1

Approximate significance of smooth terms:

	edf	Ref.df	F	p-value
s(plate)	3.694	4	11.54	$<2e^{-16}$ ***

Signif. codes: 0 '***' 0.001 '**' 0.01 '*' 0.05 '.' 0.1 ' ' 1

> pairwise.t.test(light$value, light$light_group, p.adjust.method = "bonferroni")

Pairwise comparisons using t tests with pooled SD

data: light$value and light$light_group

	dark	HighLight	Lowlight
HighLight	$5.6e^{-16}$	-	-
Lowlight	$7.3e^{-08}$	0.018	-
MediumLight	$3.0e^{-16}$	1.000	0.035

P value adjustment method: bonferroni

Results for the effect of light fluence on first peak after release:

value ~ light_group + s(plate, bs = "re")

Parametric coefficients:

	Estimate	Std. Error	t value	Pr(>|t|)
(Intercept)	3.346	1.323	2.530	0.0124 *
light_groupHighLight	13.261	1.839	7.210	$2.47e^{-11}$ ***
light_groupLowlight	4.129	1.871	2.206	0.0289 *
light_groupMediumLight	7.453	1.820	4.096	$6.82e^{-05}$ ***

Signif. codes: 0 '***' 0.001 '**' 0.01 '*' 0.05 '.' 0.1 ' ' 1

> pairwise.t.test(light$value, light$light_group, p.adjust.method = "bonferroni")

Pairwise comparisons using t tests with pooled SD

data: light$value and light$light_group

	dark	HighLight	Lowlight
HighLight	$< 2e^{-16}$	-	-
Lowlight	$5.9e^{-07}$	$< 2e^{-16}$	-
MediumLight	$< 2e^{-16}$	$1.2e^{-14}$	0.00019

P value adjustment method: bonferroni

REFERENCES AND NOTES

1. Y. M. Bar-On, R. Phillips, R. Milo, The biomass distribution on Earth. *Proc. Natl. Acad. Sci. U.S.A.* **115**, 6506–6511 (2018).

2. M. I. Soriano, B. Roibás, A. B. García, M. Espinosa-Urgel, Evidence of circadian rhythms in non-photosynthetic bacteria? *J. Circadian Rhythms* **8,** 8 (2014).

3. J. K. Paulose, J. M. Wright, A. G. Patel, V. M. Cassone, Human gut bacteria are sensitive to melatonin and express endogenous circadian rhythmicity. *PLOS ONE* **11,** e0146643 (2016).

4. J. K. Paulose, C. V. Cassone, K. B. Graniczkowska, V. M. Cassone, Entrainment of the circadian clock of the enteric bacterium *Klebsiella aerogenes* by temperature cycles. *iScience* **19**, 1202–1213 (2019).

5. Z. Eelderink-Chen, J. Bosman, F. Sartor, A. N. Dodd, Á. T. Kovács, M. Merrow, A circadian clock in a nonphotosynthetic prokaryote. *Sci. Adv.* **7**, eabe2086 (2021).

6. J. Aschoff, Exogenous and endogenous components in circadian rhythms. *Cold Spring Harb. Symp. Quant. Biol.* **25**, 11–28 (1960).

7. S. S. Branda, Å. Vik, L. Friedman, R. Kolter, Biofilms: The matrix revisited. *Trends Microbiol.* **13,** 20–26 (2005).

8. R. Gallegos-Monterrosa, E. Mhatre, Á. T. Kovács, Specific *Bacillus subtilis* 168 variants form biofilms on nutrient-rich medium. *Microbiology* **162**, 1922–1932 (2016).

9. D. B. Kearns, R. Losick, Swarming motility in undomesticated *Bacillus subtilis*. *Mol. Microbiol.* **49**, 581–590 (2003).

10. P. Stefanic, I. Mandic-Mulec, Social interactions and distribution of *Bacillus subtilis* pherotypes at microscale. *J. Bacteriol.* **191**, 1756–1764 (2009).

11. H. T. Kiesewalter, C. N. Lozano-Andrade, M. Wibowo, M. L. Strube, G. Maróti, D. Snyder, T. S. Jørgensen, T. O. Larsen, V. S. Cooper, T. Weber, Á. T. Kovács, Genomic and chemical diversity of

Bacillus subtilis secondary metabolites against plant pathogenic fungi. *mSystems* **6,** e00770–e007720 (2021).

12. J. M. Hurley, A. Dasgupta, J. M. Emerson, X. Zhou, C. S. Ringelberg, N. Knabe, A. M. Lipzen, E. A. Lindquist, C. G. Daum, K. W. Barry, I. V. Grigoriev, K. M. Smith, J. E. Galagan, D. Bell-Pedersen, M. Freitag, C. Cheng, J. J. Loros, J. C. Dunlap, Analysis of clock-regulated genes in *Neurospora* reveals widespread posttranscriptional control of metabolic potential. *Proc. Natl. Acad. Sci. U.S.A.* **111**, 16995–17002 (2014).

13. D. K. Welsh, S.-H. Yoo, A. C. Liu, J. S. Takahashi, S. A. Kay, Bioluminescence imaging of individual fibroblasts reveals persistent, independently phased circadian rhythms of clock gene expression. *Curr. Biol.* **14**, 2289–2295 (2004).

14. F. E. Belbin, Z. B. Noordally, S. J. Wetherill, K. A. Atkins, K. A. Franklin, A. N. Dodd, Integration of light and circadian signals that regulate chloroplast transcription by a nuclear-encoded sigma factor. *New Phytol.* **213**, 727–738 (2017).

15. T. Roenneberg, S. Daan, M. Merrow, The art of entrainment. *J. Biol. Rhythms* **18,** 183–194 (2003).

16. M. Ávila-Pérez, J. B. van der Steen, R. Kort, K. J. Hellingwerf, Red light activates the σ^B-mediated general stress response of *Bacillus subtilisvia* the energy branch of the upstream signaling cascade. *J. Bacteriol.* **192**, 755–762 (2010).

17. J. Aschoff, C. von Goetz, Masking of circadian activity rhythms in hamsters by darkness. *J. Comp. Physiol. A* **162**, 559–562 (1988).

18. H. G. McWatters, R. M. Bastow, A. Hall, A. J. Millar, The *ELF3 zeitnehmer* regulates light signalling to the circadian clock. *Nature* **408**, 716–720 (2000).

19. J. Rémi, M. Merrow, T. Roenneberg, A circadian surface of entrainment: Varying T, τ, and photoperiod in *Neurospora crassa*. *J. Biol. Rhythms* **25**, 318–328 (2010).

20. Y. Tan, Z. Dragovic, T. Roenneberg, M. Merrow, Entrainment dissociates transcription and translation of a circadian clock gene in *Neurospora*. *Curr. Biol.* **14**, 433–438 (2004).

21. V. G. Bruce, Environmental entrainment of circadian rhythms. *Cold Spring Harb. Symp. Quant. Biol.* **25**, 29–48 (1960).

22. M. Merrow, M. Brunner, T. Roenneberg, Assignment of circadian function for the *Neurospora* clock gene frequency. *Nature* **399,** 584–586 (1999).

23. T. Roenneberg, Z. Dragovic, M. Merrow, Demasking biological oscillators: Properties and principles of entrainment exemplified by the *Neurospora* circadian clock. *Proc. Natl. Acad. Sci. U.S.A.* **102,** 7742–7747 (2005).

24. C. S. Pittendrigh, S. Daan, A Functional analysis of circadian pacemakers in nocturnal rodents. *J. Comp. Physiol. A* **106,** 223–252 (1976).

25. C. S. Pittendrigh, Circadian rhythms and the circadian organization of living systems. *Cold Spring Harb. Symp. Quant. Biol.* **25**, 159–184 (1960).

26. D. Bates, M. Mächler, B. M. Bolker, S. C. Walker, Fitting linear mixed-effects models using lme4. *J. Stat. Softw.* **67,** 1–48 (2015).

27. M. M. Canal-Corretger, J. Vilaplana, T. Cambras, A. Díez-Noguera, Effect of light on the development of the circadian rhythm of motor activity in the mouse. *Chronobiol. Int.* **18**, 683–696 (2001).

28. C. H. Johnson, J. W. Hastings, Circadian phototransduction: Phase resetting and frequency of the circadian clock of *Gonyaulax* cells in red light. *J. Biol. Rhythms* **4**, 417–437 (1989).

29. T. Roenneberg, D. Morse, Two circadian oscillators in one cell. *Nature* **362**, 362–364 (1993).

30. M. L. Pay, D. W. Kim, D. E. Somers, J. K. Kim, M. Foo, Modelling of plant circadian clock for characterizing hypocotyl growth under different light quality conditions. *In Silico Plants* **4**, diac001 (2022).

31. T. Ohara, H. Fukuda, I. T. Tokuda, An extended mathematical model for reproducing the phase response of *Arabidopsis thaliana* under various light conditions. *J. Theor. Biol.* **382**, 337–344 (2015).

32. T. Woelders, E. J. Wams, M. C. M. Gordijn, D. G. M. Beersma, R. A. Hut, Integration of color and intensity increases time signal stability for the human circadian system when sunlight is obscured by clouds. *Sci. Rep.* **8**, 15214 (2018).

33. H. G. Nimmo, Entrainment of *Arabidopsis* roots to the light:dark cycle by light piping. *Plant Cell Environ.* **41**, 1742–1748 (2018).

34. J. T. Woolley, E. W. Stoller, Light penetration and light-induced seed germination in soil. *Plant Physiol.* **61**, 597–600 (1978).

35. Y. Ouyang, C. R. Andersson, T. Kondo, S. S. Golden, C. H. Johnson, Resonating circadian clocks enhance fitness in cyanobacteria. *Proc. Natl. Acad. Sci. U.S.A.* **95**, 8660–8664 (1998).

36. A. N. Dodd, N. Salathia, A. Hall, E. Kévei, R. Tóth, F. Nagy, J. M. Hibberd, A. J. Millar, A. A. R. Webb, Plant circadian clocks increase photosynthesis, growth, survival, and competitive advantage. *Science* **309**, 630–633 (2005).

37. S. Daan, K. Spoelstra, U. Albrecht, I. Schmutz, M. Daan, B. Daan, F. Rienks, I. Poletaeva, G. Dell'Omo, A. Vyssotski, H.-P. Lipp, Lab mice in the field: Unorthodox daily activity and effects of a dysfunctional circadian clock allele. *J. Biol. Rhythms* **26**, 118–129 (2011).

38. A. Newman, E. Picot, S. Davies, S. Hilton, I. A. Carré, G. D. Bending, Circadian rhythms in the plant host influence rhythmicity of rhizosphere microbiota. *BMC Biol.* **20,** 235 (2022).

39. M. Ávila-Pérez, K. J. Hellingwerf, R. Kort, Blue light activates the σ^B-dependent stress response of *Bacillus subtilis* via YtvA. *J. Bacteriol.* **188**, 6411–6414 (2006).

40. T. A. Gaidenko, T. J. Kim, A. L. Weigel, M. S. Brody, C. W. Price, The blue-light receptor YtvA acts in the environmental stress signaling pathway of *Bacillus subtilis*. *J. Bacteriol.* **188**, 6387–6395 (2006).

41. S. Akbar, T. A. Gaidenko, C. Min Kang, M. O'Reilly, K. M. Devine, C. W. Price, New family of regulators in the environmental signaling pathway which activates the general stress transcription factor ς^B of *Bacillus subtilis*. *J. Bacteriol.* **183**, 1329–1338 (2001).

42. M. Avila-Pérez, J. Vreede, Y. Tang, O. Bende, A. Losi, W. Gärtner, K. Hellingwerf, *In vivo* mutational analysis of YtvA from *Bacillus subtilis*: Mechanism of light activation of the general stress response. *J. Biol. Chem.* **284**, 24958–24964 (2009).

43. R. S. Edgar, E. W. Green, Y. Zhao, G. Van Ooijen, M. Olmedo, X. Qin, Y. Xu, M. Pan, U. K. Valekunja, K. A. Feeney, E. S. Maywood, M. H. Hastings, N. S. Baliga, M. Merrow, A. J. Millar, C. H. Johnson, C. P. Kyriacou, J. S. O'Neill, A. B. Reddy, Peroxiredoxins are conserved markers of circadian rhythms. *Nature* **485**, 459–464 (2012).

44. A. G. Albrecht, D. J. A. Netz, M. Miethke, A. J. Pierik, O. Burghaus, F. Peuckert, R. Lill, M. A. Marahiel, SufU is an essential iron-sulfur cluster scaffold protein in *Bacillus subtilis*. *J. Bacteriol.* **192**, 1643–1651 (2010).

45. Y. A. Oñate, S. J. Vollmer, R. L. Switzer, M. K. Johnson, Spectroscopic characterization of the iron-sulfur cluster in *Bacillus subtilis* glutamine phosphoribosylpyrophosphate amidotransferase. *J. Biol. Chem.* **264**, 18386–18391 (1989).

46. M M. Blahut, E. Sanchez, C. E. Fisher, F. W. Outten, Fe-S cluster biogenesis by the bacterial Suf pathway. *Biochim. Biophys. Acta Mol. Cell. Res.* **1867,** 118829 (2020).

47. L. Hederstedt, A. Lewin, M. Throne-Holst, Heme A synthase enzyme functions dissected by mutagenesis of *Bacillus subtilis* CtaA. *J. Bacteriol.* **187**, 8361–8369 (2005).

48. N. B. Ivleva, M. R. Bramlett, P. A. Lindahl, S. S. Golden, LdpA: A component of the circadian clock senses redox state of the cell. *EMBO J.* **24**, 1202–1210 (2005).

49. M. J. Rust, S. S. Golden, E. K. O'Shea, Light-driven changes in energy metabolism directly entrain the cyanobacterial circadian oscillator. *Science* **331**, 220–223 (2011).

50. D. B. Pedrolli, C. Kühm, D. C. Sévin, M. P. Vockenhuber, U. Sauer, B. Suess, M. Mack, A dual control mechanism synchronizes riboflavin and sulphur metabolism in *Bacillus subtilis*. *Proc. Natl. Acad. Sci. U.S.A.* **112**, 14054–14059 (2015).

51. A. B. James, J. A. Monrea, G. A. Nimmo, C. L. Kelly, P. Herzyk, G. I. Jenkins, H. G. Nimmo, The circadian clock in *Arabidopsis* roots is a simplified slave version of the clock in shoots. *Science* **322**, 1832–1835 (2008).

52. M. Endo, H. Shimizu, M. A. Nohales, T. Araki, S. A. Kay, Tissue-specific clocks in *Arabidopsis* show asymmetric coupling. *Nature* **515**, 419–422 (2014).

53. C. Schmal, E. D. Herzog, H. Herzel, Measuring relative coupling strength in circadian systems. *J. Biol. Rhythms* **33**, 84–98 (2018).

54. C. J. Guenthner, M. E. Luitje, L. A. Pyle, P. C. Molyneux, J. K. Yu, A. S. Li, T. L. Leise, M. E. Harrington, Circadian rhythms of PER2::LUC in individual primary mouse hepatocytes and cultures. *PLOS ONE* **9,** e87573 (2014).

55. Y. Hirata, R. Enoki, K. Kuribayashi-Shigetomi, Y. Oda, S. Honma, K.-IHonma, Circadian rhythms in *Per1*, PER2 and Ca^{2+} of a solitary SCN neuron cultured on a microisland. *Sci. Rep.* **9**, 18271 (2019).

56. A. Frank, C. C. Matiolli, A. J. C. Viana, T. J. Hearn, J. Kusakina, F. E. Belbin, D. Wells Newman, A. Yochikawa, D. L. Cano-Ramirez, A. Chembath, K. Cragg-Barber, M. J. Haydon, C. T. Hotta, M. Vincentz, A. A. R. Webb, A. N. Dodd, Circadian entrainment in *Arabidopsis* by the sugar-responsive transcription factor bZIP63. *Curr. Biol.* **28**, 2597–2606.e6 (2018).

57. E. S. Schernhammer, F. Laden, F. E. Speizer, W. C. Willett, D. J. Hunter, I. Kawachi, G. A. Colditz, Rotating night shifts and risk of breast cancer in women participating in the Nurses' Health Study. *J. Natl. Cancer Inst.* **93,** 1563–1568 (2001).

58. E. S. Schernhammer, F. Laden, F. E. Speizer, W. C. Willett, D. J. Hunter, I. Kawachi, C. S. Fuchs, G. A. Colditz, Night-shift work and risk of colorectal cancer in the Nurses' Health Study. *J. Natl. Cancer Inst.* **95,** 825–828 (2003).

59. T. Roenneberg, M. Merrow, Molecular circadian oscillators: An alternative hypothesis. *J. Biol. Rhythms* **13**, 167–179 (1998).

60. D. W. Kim, C. Chang, X. Chen, A. C. Doran, F. Gaudreault, T. Wager, G. J. DeMarco, J. K. Kim, Systems approach reveals photosensitivity and PER 2 level as determinants of clock-modulator efficacy. *Mol. Syst. Biol.* **15**, e8838 (2019).

61. J. K. Kim, D. B. Forger, M. Marconi, D. Wood, A. Doran, T. Wager, C. Chang, K. M. Walton, Modeling and validating chronic pharmacological manipulation of circadian rhythms. *CPT Pharmacometrics Syst. Pharmacol.* **2**, e57 (2013).

62. P. Fortnagel, E. Freese, Analysis of sporulation mutants. II. Mutants blocked in the citric acid cycle. *J. Bacteriol.* **95**, 1431–1438 (1968).

63. T. Zielinski, A. M. Moore, E. Troup, K. J. Halliday, A. J. Millar, Strengths and limitations of period estimation methods for circadian data. *PLOS ONE* **9,** e96462 (2014).

64. A. Díez-Noguera, Methods for serial analysis of long time series in the study of biological rhythms. *J. Circadian Rhythms* **11**, 7 (2014).

65. H. Wickham H, *ggplot2: Elegant Graphics for Data Analysis*. (Springer-Verlag New York, 2016).

66. B.-M. Koo, G. Kritikos, J. D. Farelli, H. Todor, K. Tong, H. Kimsey, I. Wapinski, M. Galardini, A. Cabal, J. M. Peters, A.-B. Hachmann, D. Z. Rudner, K. N. Allen, A. Typas, C. A. Gross, Construction and analysis of two genome-scale deletion libraries for *Bacillus subtilis*. *Cell Syst.* **4**, 291–305.e7 (2017).

67. I. Seccareccia, Á. T. Kovács, R. Gallegos-Monterrosa, M. Nett, Unraveling the predator-prey relationship of *Cupriavidus necator* and *Bacillus subtilis*. *Microbiol. Res.* **192**, 231–238 (2016).

68. Á. T. Kovács, M. van Hartskamp, O. P. Kuipers, R. van Kranenburg, Genetic tool development for a new host for biotechnology, the thermotolerant bacterium *Bacillus coagulans*. *Appl. Environ. Microbiol.* **76**, 4085–4088 (2010).

69. J. Husse, A. Leliavski, A. H. Tsang, H. Oster, G. Eichele, The light-dark cycle controls peripheral rhythmicity in mice with a genetically ablated suprachiasmatic nucleus clock. *FASEB J.* **28**, 4950–4960 (2014).

70. S. J. Aton, G. D. Block, H. Tei, S. Yamazaki, E. D. Herzog, Plasticity of circadian behavior and the suprachiasmatic nucleus following exposure to non-24-hour light cycles. *J. Biol. Rhythms* **19**, 198–207 (2004).

71. P. C. Molyneux, M. K. Dahlgren, M. E. Harrington, Circadian entrainment aftereffects in suprachiasmatic nuclei and peripheral tissues in vitro. *Brain Res.* **1228**, 127–134 (2008).

72. N. Mrosovsky, Aschoff's rule in retinally degenerate mice. *J. Comp. Physiol. A Neuroethol. Sens. Neural Behav. Physiol.* **189**, 75–78 (2003).

73. S. Steinlechner, B. Jacobmeier, F. Scherbarth, H. Dernbach, F. Kruse, U. Albrecht, Robust circadian rhythmicity of *Per1* and *Per2* mutant mice in constant light, and dynamics of *Per1* and *Per2* gene expression under long and short photoperiods. *J. Biol. Rhythms* **17**, 202–209 (2002).

74. B. Thines, F. G. Harmon, Ambient temperature response establishes ELF3 as a required component of the core *Arabidopsis* circadian clock. *Proc. Natl. Acad. Sci. U.S.A.* **107**, 3257–3262 (2010).

75. E. Kolmos, E. Herrero, N. Bujdoso, A. J. Millar, R. Tóth, P. Gyula, F. Nagy, S. J. Davis, A reduced-function allele reveals that EARLY FLOWERING3 repressive action on the circadian clock is modulated by phytochrome signals in *Arabidopsis*. *Plant Cell* **23**, 3230–3246 (2011).

76. A. N. Dodd, N. Dalchau, M. J. Gardner, S. J. Baek, A. A. R. Webb, The circadian clock has transient plasticity of period and is required for timing of nocturnal processes in *Arabidopsis*. *New Phytol.* **201**, 168–179 (2014).

77. D. E. Somers, P. F. Devlin, S. A. Kay, Phytochromes and cryptochromes in the entrainment of the *Arabidopsis* circadian clock. *Science* **282**, 1488–1490 (1998).

78. Y. He, Y. Yu, X. Wang, Y. Qin, C. Su, L. Wang, Aschoff's rule on circadian rhythms orchestrated by blue light sensor CRY2 and clock component PRR9. *Nat. Commun.* **13**, 5869 (2022).

79. J. Diegmann, A. Stück, C. Madeti, T. Roenneberg, Entrainment elicits period aftereffects in *Neurospora crassa*. *Chronobiol. Int.* **27**, 1335–1347 (2010).

80. K. Schneider, S. Perrino, K. Oelhafen, S. Li, A. Zatsepin, P. Lakin-Thomas, S. Brody, Rhythmic conidiation in constant light in *Vivid* mutants of *Neurospora crassa*. *Genetics* **181,** 917–931 (2009).

81. T. Roenneberg, G. N. Colfax, J. W. Hastings, A circadian rhythm of population behavior in *Gonyaulax polyedra*. *J. Biol. Rhythms* **4**, 201–216 (1989).

82. T. Roenneberg, J. W. Hastings, Are the effects of light on phase and period of the *Gonyaulax* clock mediated by different pathways? *Photochem. Photobiol.* **53**, 525–533 (1991).

83. T. Roenneberg, J. W. Hastings, Two photoreceptors control the circadian clock of a unicellular alga. *Naturwissenschaften* **75**, 206–207 (1988).

84. J. Toepel, J. E. McDermott, T. C. Summerfield, L. A. Sherman, Transcriptional analysis of the unicellular, diazotrophic cyanobacterium *Cyanothece* sp. ATCC 51142 grown under short day/night cycles. *J. Phycol.* **45**, 610–620 (2009).

85. T. Yoshida, Y. Murayama, H. Ito, H. Kageyama, T. Kondo, Nonparametric entrainment of the in vitro circadian phosphorylation rhythm of cyanobacterial KaiC by temperature cycle. *Proc. Natl. Acad. Sci. U.S.A.* **106**, 1648–1653 (2009).

86. S. R. Mackey, J. L. Ditty, E. M. Clerico, S. S. Golden, Detection of rhythmic bioluminescence from luciferase reporters in cyanobacteria. *Methods Mol. Biol.* **362**, 115–129 (2007).

87. M. Katayama, T. Kondo, J. Xiong, S. S. Golden, *ldpA* encodes an iron-sulfur protein involved in light-dependent modulation of the circadian period in the cyanobacterium *Synechococcus elongatus* PCC 7942. *J. Bacteriol.* **185**, 1415–1422 (2003).

88. R Core Team, R: A language and environment for statistical computing. (R Foundation for Statistical Computing, 2021). https://www.R-project.org/.
